# Supplementary material for: Impact of Energy and Protein Delivery to Critically Ill Patients: A Systematic Review and Meta-Analysis of Randomized Controlled Trials
Source: Nutrients. 2022 Nov 16;14(22):4849. doi: 10.3390/nu14224849 (PMC9698683; doi:10.3390/nu14224849)
Supplement: Supplementary file 1 [file nutrients-14-04849-s001.zip › nutrients-2021546-supplementary.pdf]

Supplemental file

Impact of energy and protein delivery to critically ill patients: A systematic review and meta-analysis of randomized controlled trials

Contents

1. Search Strategy……………...……………………………………………………………….2
2. Risk of bias and effect on outcomes about optimal energy delivery…………….…………..7
3. Risk of bias and effect on outcomes about optimal protein delivery………………………..30
4. Evidence profile about optimal energy delivery……………………………………………..43
5. Evidence profile about optimal protein delivery…………………………………………….45

1. Search strategy

Data base

- MEDLINE
- CENTRAL
- Web of Science

MEDLINE (via PubMed) search strategy（Searched in AM–12:55, November 2, 2021）

	Search formula	Results
#1	"intensive care units"[MeSH Terms]	94,967
#2	"critical illness"[MeSH Terms]	33,859
#3	"critical care"[MeSH Terms]	62,323
#4	"intensive care"[Title/Abstract]	166,568
#5	"icu"[Title/Abstract]	68,328
#6	"critical ill*"[Title/Abstract]	12,014
#7	"critically ill*"[Title/Abstract]	51,609
#8	"critical care"[Title/Abstract]	36,374
#9	#1 OR #2 OR #3 OR #4 OR #5 OR #6 OR #7 OR #8	291,073
#10	"nutritional support"[MeSH Terms]	47,208
#11	"enteral nutrition"[Title/Abstract]	9,794
#12	"parenteral nutrition"[Title/Abstract]	21,375
#13	"enteral feeding"[Title/Abstract]	5,723
#14	#10 OR #11 OR #12 OR #13	58,023
#15	"dietary proteins"[MeSH Terms]	102,686

#16	"amino acids"[MeSH Terms]	827,170
#17	"protein*"[Title/Abstract]	3,264,817
#18	"Energy Intake*"[MeSH Terms]	48,965
#19	"energy"[Title/Abstract]	712,225
#20	"trophic"[Title/Abstract]	30,472
#21	"hypocaloric"[Title/Abstract]	1,941
#22	"full feeding"[Title/Abstract]	164
#23	"underfeeding"[Title/Abstract]	712
#24	#15 OR #16 OR #17 OR #18 OR #19 OR #20 OR #21 OR #22 OR #23	4,520,841
#25	#14 AND #24	13,688
#26	(randomized controlled trial[pt] OR controlled clinical trial[pt] OR randomized[tiab] OR placebo[tiab] OR drug therapy[sh] OR randomly[tiab] OR trial[tiab] OR groups[tiab] NOT (animals [mh] NOT humans [mh]))	4,554,553
#27	#9 AND #25 AND #26	1,031

Cochrane Central Register of Controlled Trials search strategy (Searched in PM–4:25, November 3, 2021)

#1	MeSH descriptor: [Intensive Care Units] explode all trees	3864
#2	MeSH descriptor: [Critical Illness] explode all trees	2466
#3	MeSH descriptor: [Critical Care] explode all trees	2157
#4	("intensive care"):ti,ab,kw	25378
#5	("icu"):ti,ab,kw	14244

#6	("critical ill*"):ti,ab,kw	97
#7	("critically ill*"):ti,ab,kw	7413
#8	("critical care"):ti,ab,kw	4200
#9	#1 OR #2 OR #3 OR #4 OR #5 OR #6 OR #7 OR #8	35943
#10	MeSH descriptor: [Nutritional Support] explode all trees	3510
#11	("enteral nutrition"):ti,ab,kw	4569
#12	("parenteral nutrition"):ti,ab,kw	4239
#13	("enteral feeding"):ti,ab,kw	1482
#14	#10 OR #11 OR #12 OR #13	8643
#15	MeSH descriptor: [Dietary Proteins] explode all trees	4292
#16	MeSH descriptor: [Amino Acids] explode all trees	22199
#17	("protein*"):ti,ab,kw	84217
#18	MeSH descriptor: [Energy Intake] explode all trees	5656
#19	(“energy”):ti,ab,kw	36,870
#20	("trophic"):ti,ab,kw	415
#21	("hypocaloric"):ti,ab,kw	1146
#22	("full feeding"):ti,ab,kw	87
#23	("underfeeding"):ti,ab,kw	85
#24	#15 OR #16 OR #17 OR #18 OR #19 OR #20 OR #21 OR #22 OR #23	134037
#25	#14 AND #24	3151
#31	#9 AND #25	912

Web of Science search strategy (Searched in AM–1:13, November 2, 2021)

#1	(ICU/TH or ICU/TA) and (PT=会議録除く)	51,885
#2	(危篤/TH or 危篤/TA) and (PT=会議録除く)	194
#3	(クリティカルケア/TH or クリティカルケア/TA) and (PT=会議録除く)	6,958
#4	#1 or #2 or #3	57,043
#5	(栄養補助/TH or 栄養補助/TA) and (PT=会議録除く)	22,173
#6	(経腸栄養/TA) and (PT=会議録除く)	6,632
#7	(静脈栄養/TA) and (PT=会議録除く)	5,232
#8	#5 or #6 or #7	25,534
#9	(食品中のタンパク質/TH or 食品中のタンパク質/TA) and (PT=会議録除く)	5,972
#10	("Amino Acids"/TH or "Amino Acids"/TA) and (PT=会議録除く)	52,993
#11	(タンパク質/TH or タンパク質/TA) and (PT=会議録除く)	614,546
#12	(タンパク質摂取量/TA) and (PT=会議録除く)	88
#13	(たんぱく質補給/TA) and (PT=会議録除く)	4
#14	(エネルギー摂取量/TH or エネルギー摂取量/TA) and (PT=会議録除く)	7,795
#15	(エネルギー/TA) and (PT=会議録除く)	20,580
#16	(trophic/TA) and (PT=会議録除く)	2,313
#17	(hypocaloric/TA) and (PT=会議録除く)	6
#18	(full/AL and (給餌/TH or feeding/TA)) and (PT=会議録除く)	22
#19	(underfeeding/TA) and (PT=会議録除く)	27
#20	#9 or #10 or #11 or #12 or #13 or #14 or #15 or #16 or #17 or #18 or #19	670,165

#21	#8 and #20	4,638
#22	ランダム化比較試験/TH or 準ランダム化比較試験/TH or ランダム化/AL or 無作為化/AL or 比較試験/AL or 臨床試験/AL or プラセボ/AL or 対照/AL or コント/AL and ロール/AL or 臨床研究/AL	142,934
#23	#4 and #21 and #22	13

2. Risk of bias and effect on outcomes about optimal energy delivery

Energy: Risk of bias summary and graph

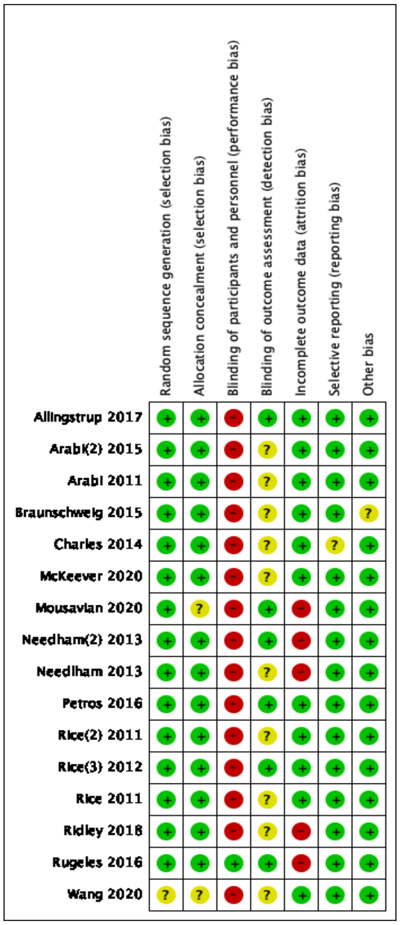

Figure S1. Risk of bias summary: review authors' judgements about each risk of bias item for each included study.

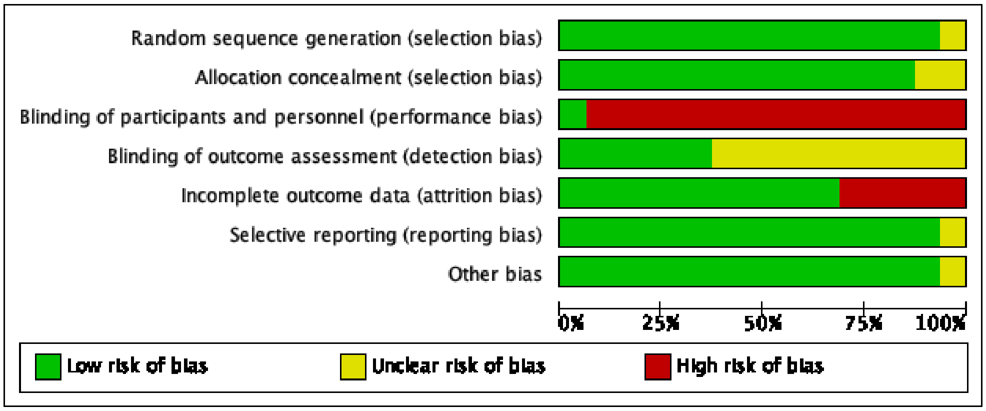

Figure S2. Risk of bias graph: review authors' judgements about each risk of bias item presented as percentages across all included studies.

Energy: Physical functions at the hospital discharge or one year after the hospital discharge

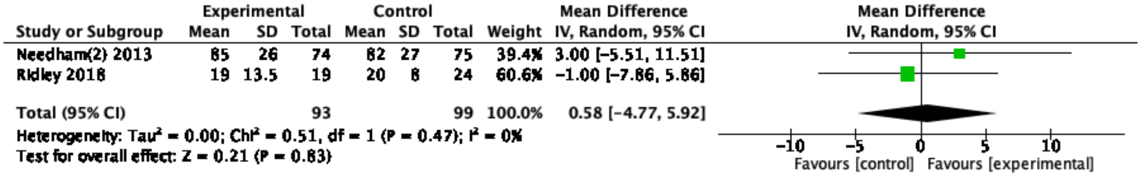

Study or Subgroup	Experimental Mean	Experimental SD	Experimental Total	Control Mean	Control SD	Control Total	Weight	Mean Difference IV, Random, 95% CI
Needham(2) 2013	85	26	74	82	27	75	39.4%	3.00 [−5.51, 11.51]
Ridley 2018	19	13.5	19	20	8	24	60.6%	−1.00 [−7.86, 5.86]
Total (95% CI)			93			99	100.0%	0.58 [−4.77, 5.92]

Heterogeneity: $Tau^2 = 0.00$; $Chi^2 = 0.51$, df = 1 (P = 0.47); $I^2 = 0\%$
Test for overall effect: Z = 0.21 (P = 0.83)

Figure S3. Forest plot of comparison: Handgrip strength.

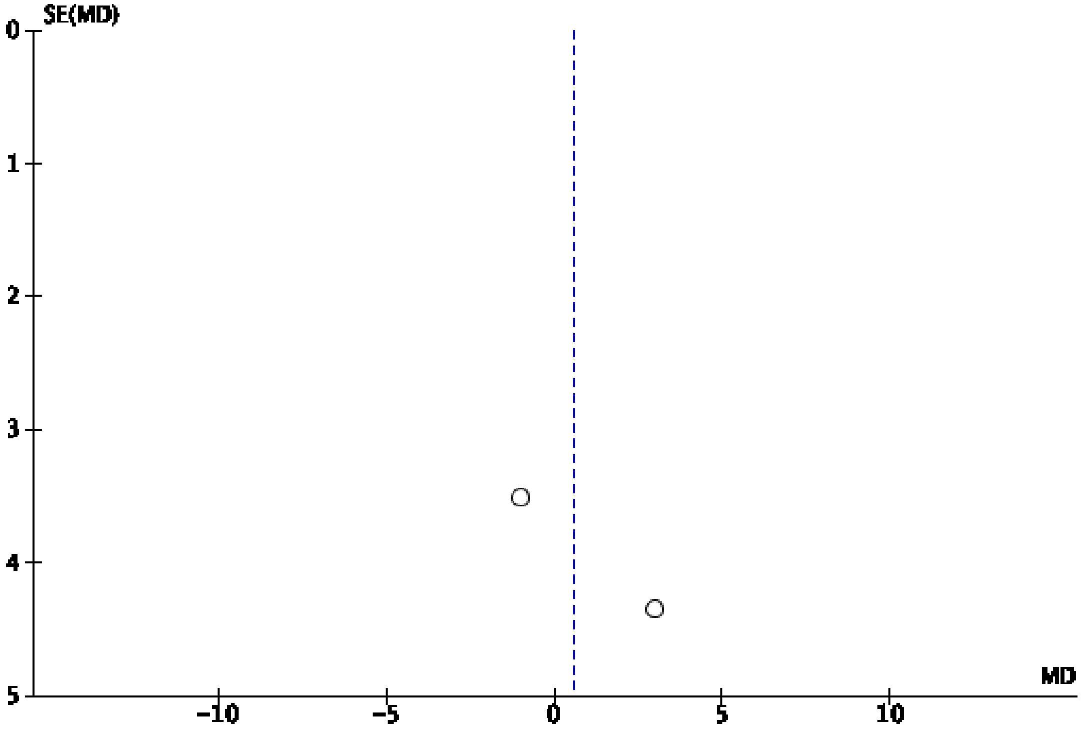

Figure S4. Funnel plot of comparison: Handgrip strength.

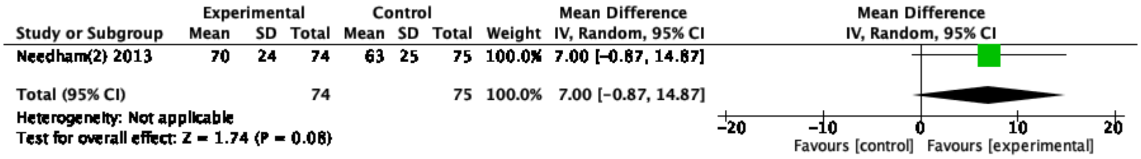

Figure S5. Forest plot of comparison: 6-minute walk distance (% prediction).

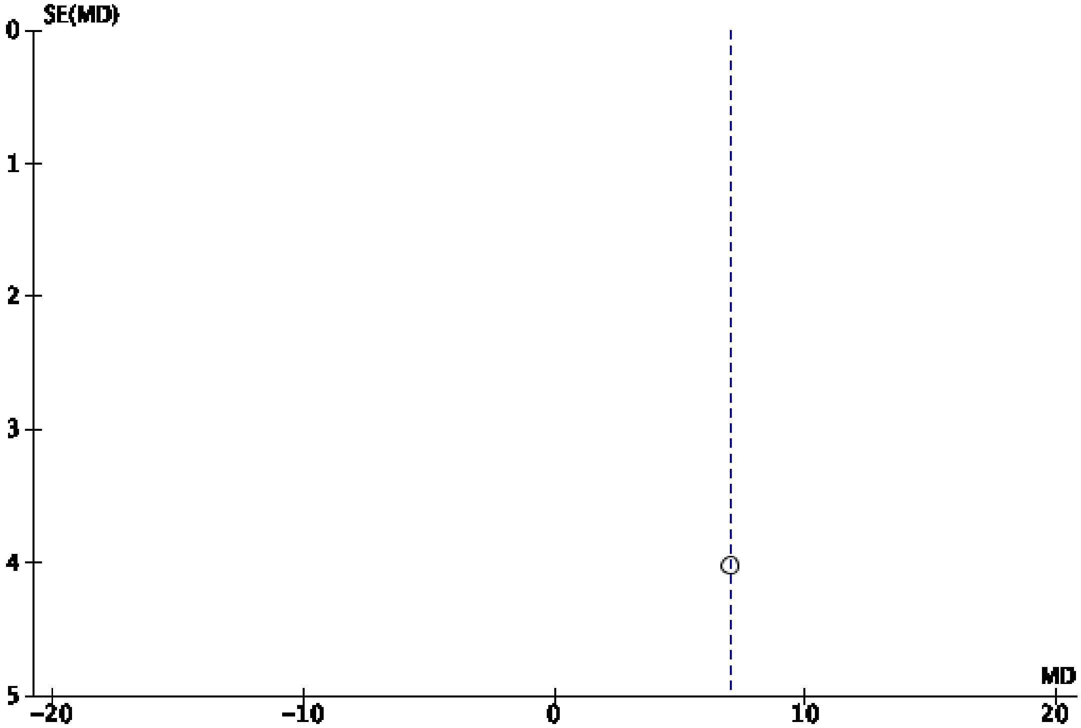

Figure S6. Funnel plot of comparison: 6-minute walk distance (% prediction).

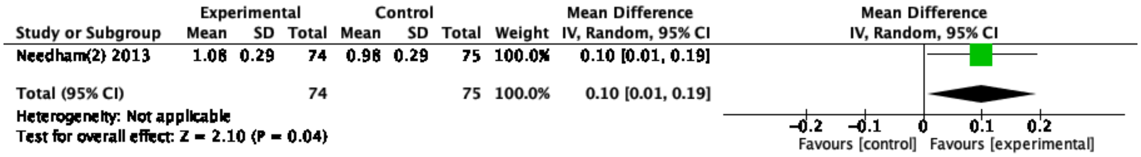

Figure S7. Forest plot of comparison: 4-minutes timed walk speed (m/s).

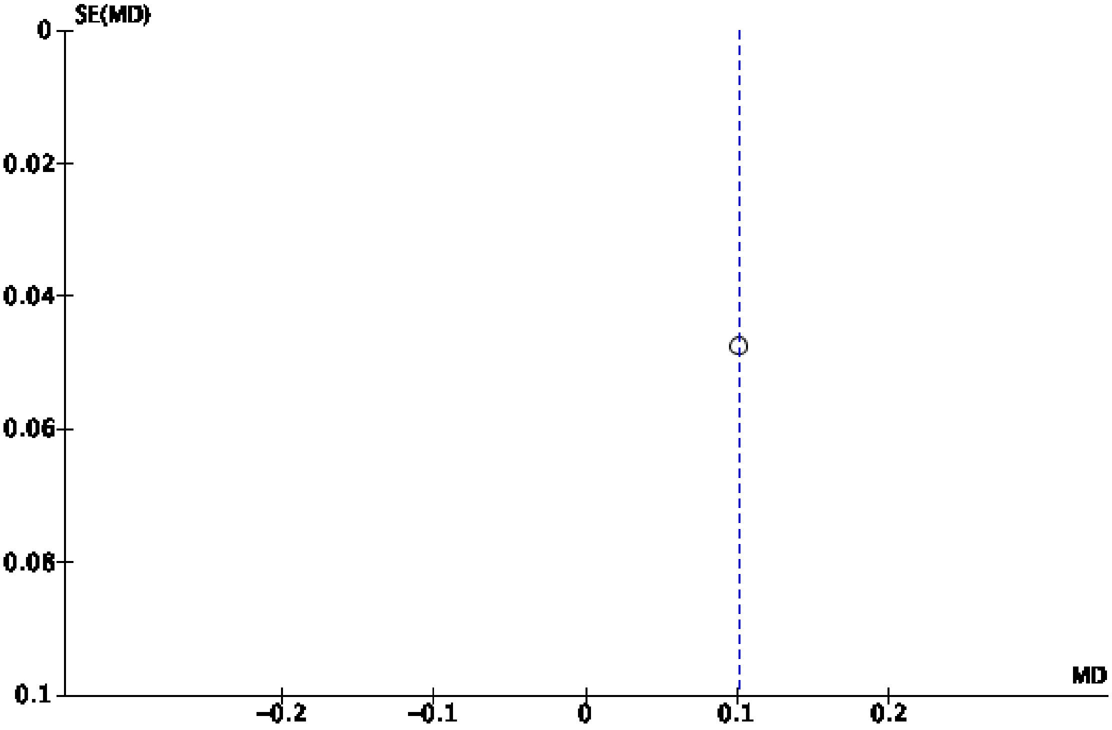

Figure S8. Funnel plot of comparison: 4-minutes timed walk speed (m/s).

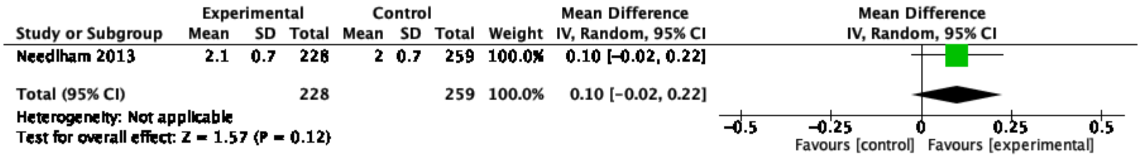

Figure S9. Forest plot of comparison: Functional performance inventory (FPI) over all.

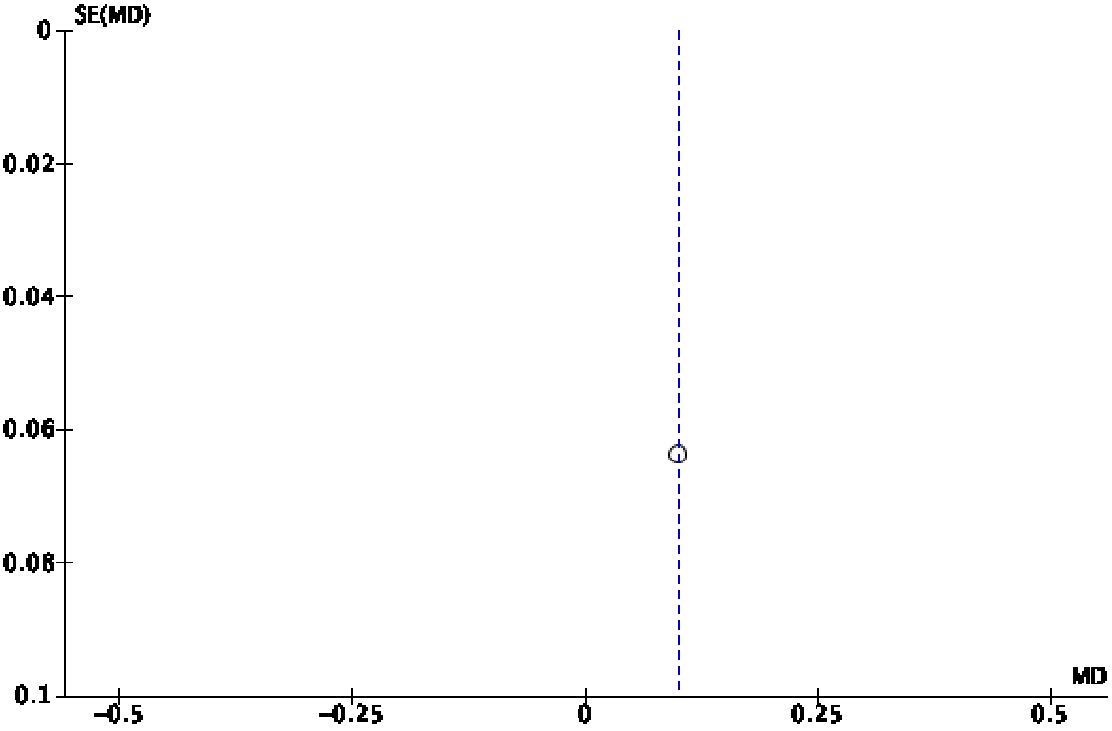

Figure S10. Funnel plot of comparison: Functional performance inventory (FPI) over all.

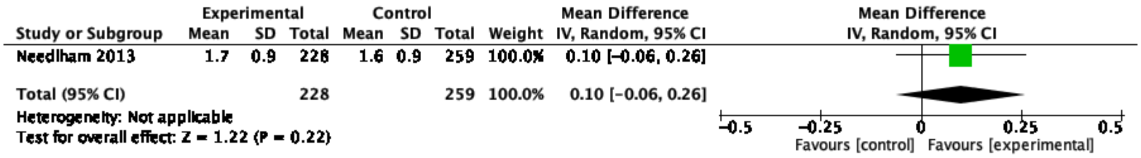

Study or Subgroup	Experimental Mean	Experimental SD	Experimental Total	Control Mean	Control SD	Control Total	Weight	Mean Difference IV, Random, 95% CI
Needlham 2013	1.7	0.9	228	1.6	0.9	259	100.0%	0.10 [−0.06, 0.26]
Total (95% CI)			228			259	100.0%	0.10 [−0.06, 0.26]
Heterogeneity: Not applicable								
Test for overall effect: Z = 1.22 (P = 0.22)								

Figure S11. Forest plot of comparison: Functional performance inventory (FPI) physical exercise.

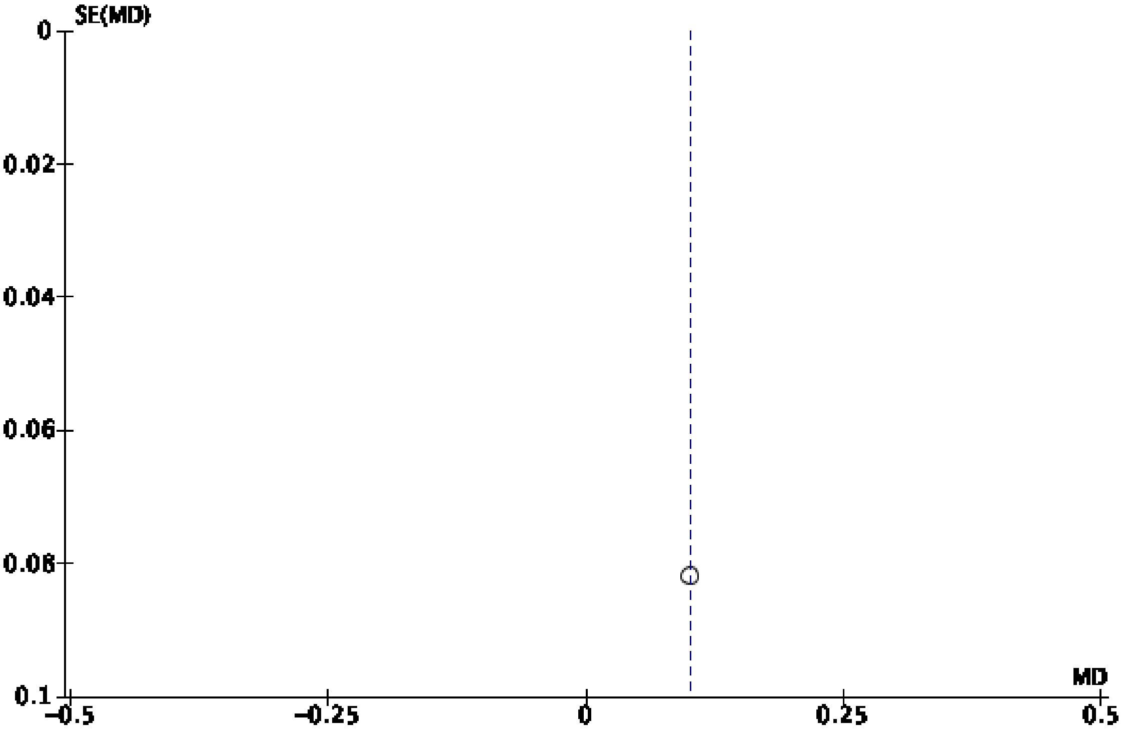

Figure S12. Funnel plot of comparison: Functional performance inventory (FPI) physical exercise.

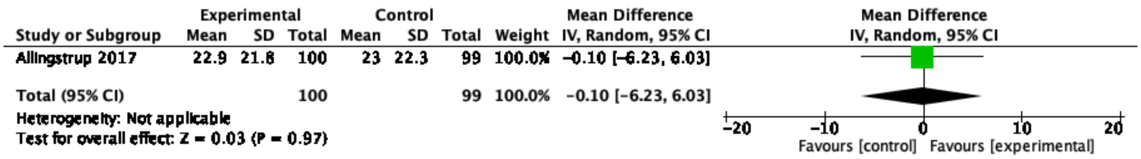

Figure S13. Forest plot of comparison: Physical component summary (PCS).

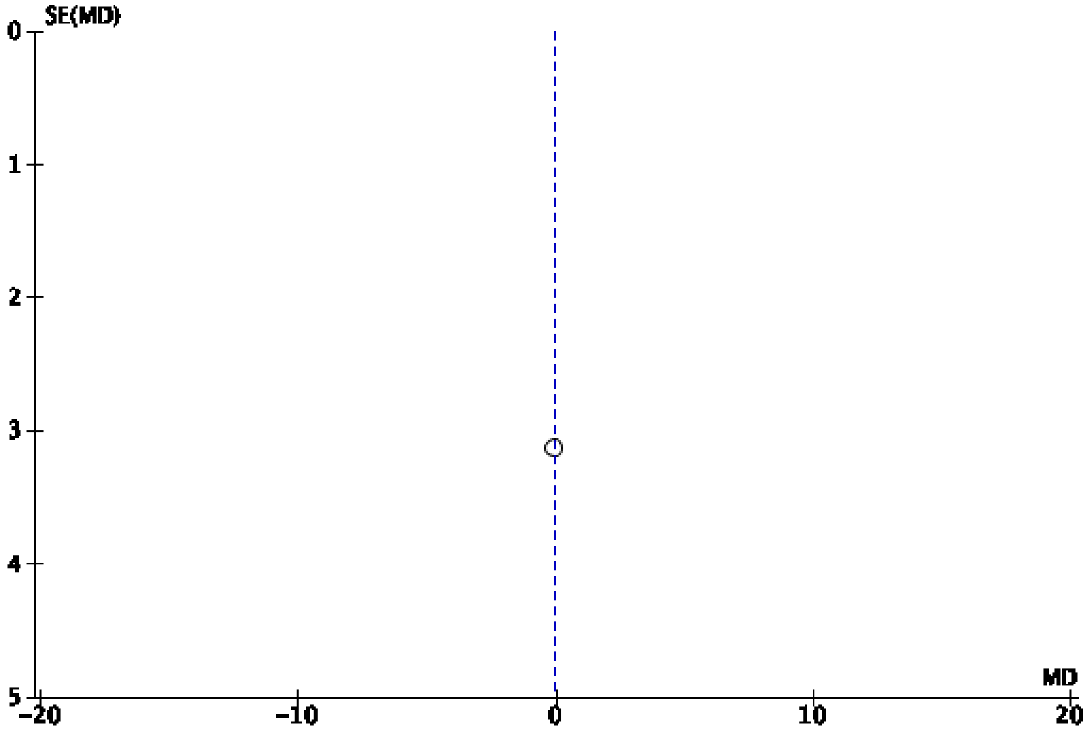

Figure S14. Funnel plot of comparison: Physical component summary (PCS).

Energy: QOL score at the hospital discharge or one year after the hospital discharge

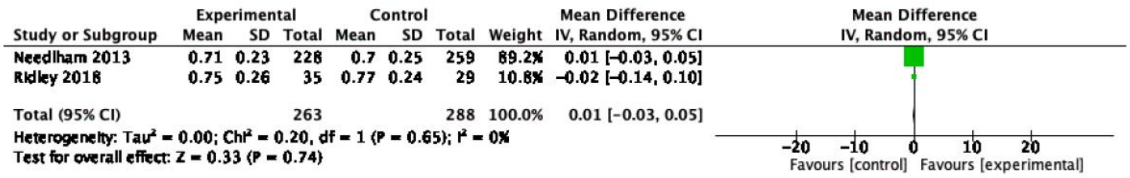

Figure S15. Forest plot of comparison: EQ-5D-3L at the hospital discharge.

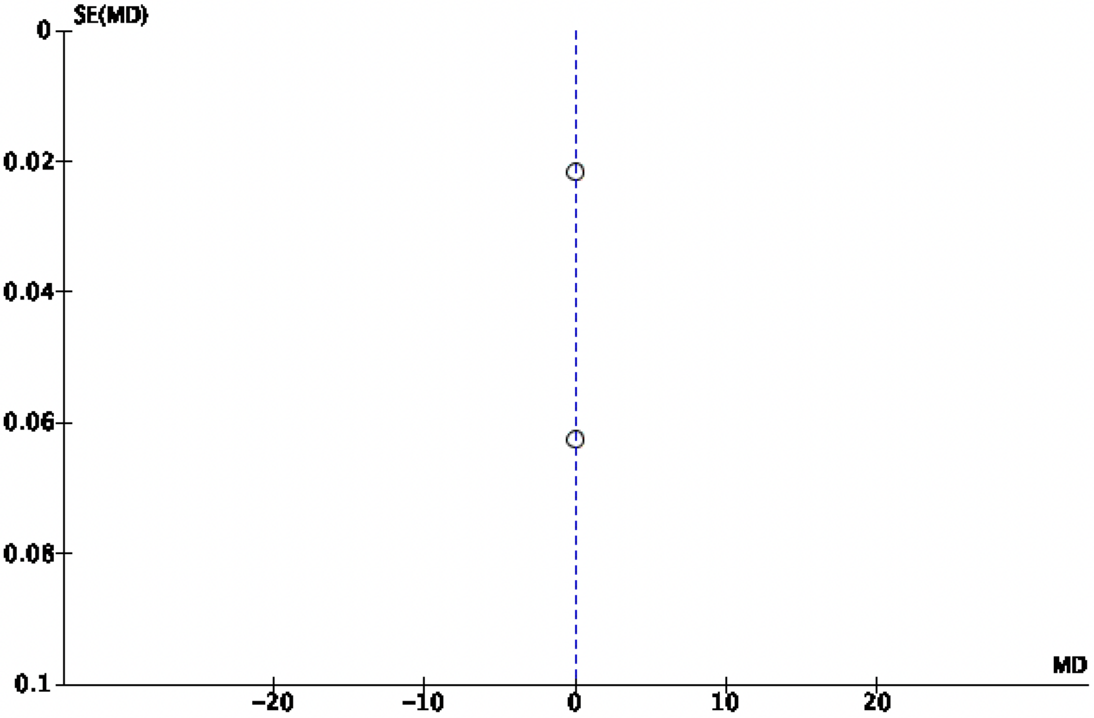

Figure S16. Funnel plot of comparison: EQ-5D-3L at the hospital discharge.

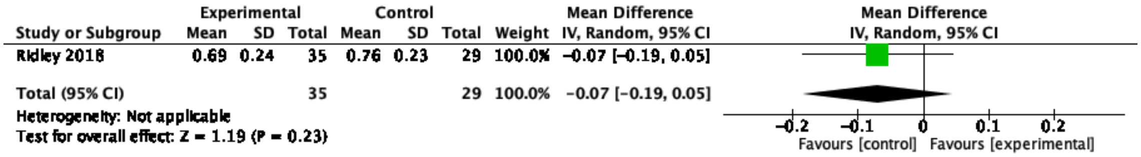

Figure S17. Forest plot of comparison: EQ-5D-3L at 90 days.

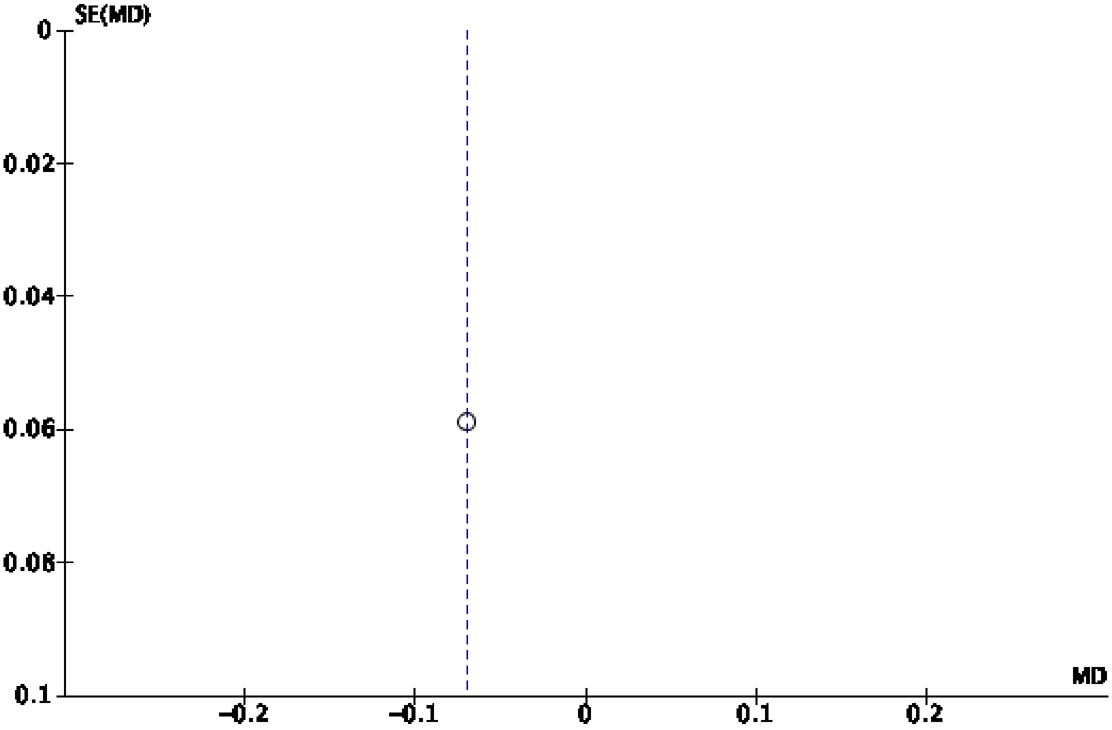

Figure S18. Funnel plot of comparison: EQ-5D-3L at 90 days.

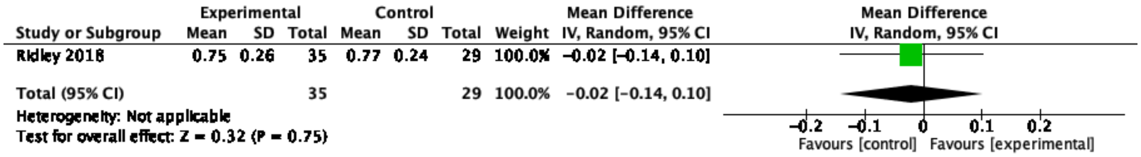

Study or Subgroup	Experimental Mean	Experimental SD	Experimental Total	Control Mean	Control SD	Control Total	Weight	Mean Difference IV, Random, 95% CI
Ridley 2018	0.75	0.26	35	0.77	0.24	29	100.0%	−0.02 [−0.14, 0.10]
Total (95% CI)			35			29	100.0%	−0.02 [−0.14, 0.10]

Heterogeneity: Not applicable
Test for overall effect: Z = 0.32 (P = 0.75)

Figure S19. Forest plot of comparison: EQ-5D-3L at 180 days.

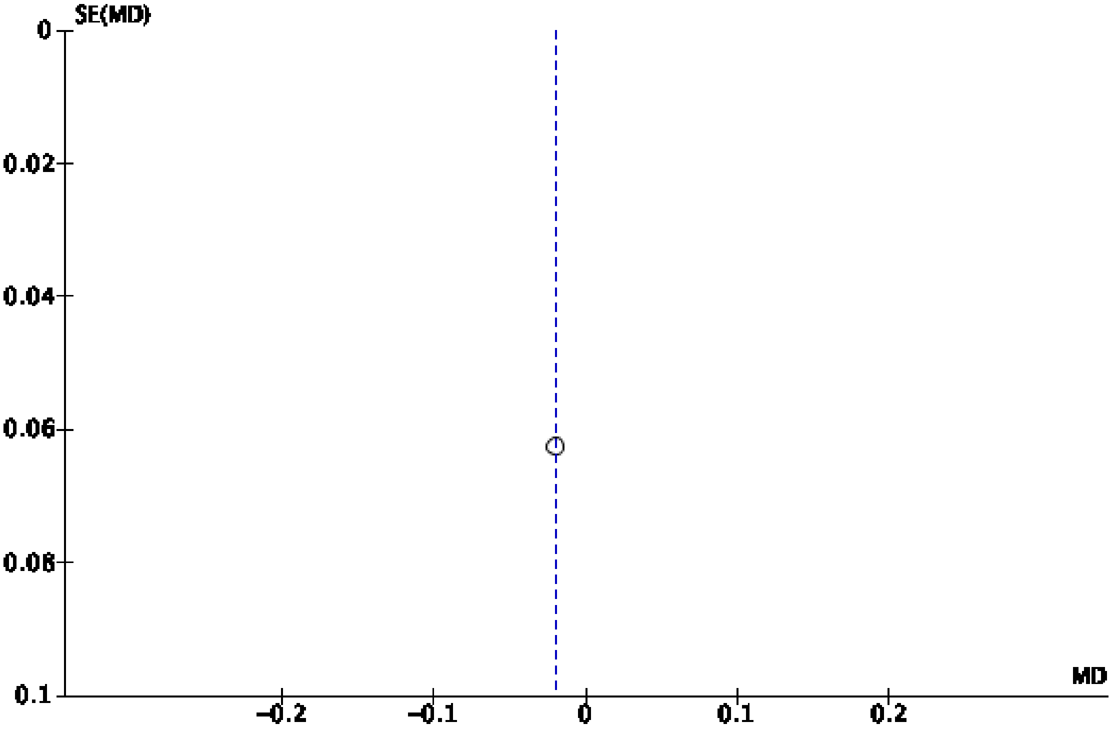

Figure S20. Funnel plot of comparison: EQ-5D-3L at 180 days.

Study or Subgroup	Experimental Mean	Experimental SD	Experimental Total	Control Mean	Control SD	Control Total	Weight	Mean Difference IV, Random, 95% CI
Needlham 2013	55	31	228	55	33	259	100.0%	0.00 [−5.69, 5.69]
Total (95% CI)			228			259	100.0%	0.00 [−5.69, 5.69]
Heterogeneity: Not applicable								
Test for overall effect: Z = 0.00 (P = 1.00)								

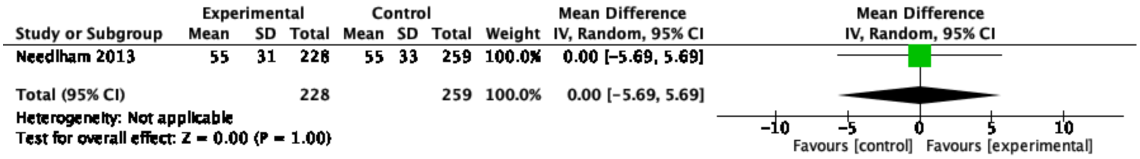

Figure S21. Forest plot of comparison: SF-36 physical function.

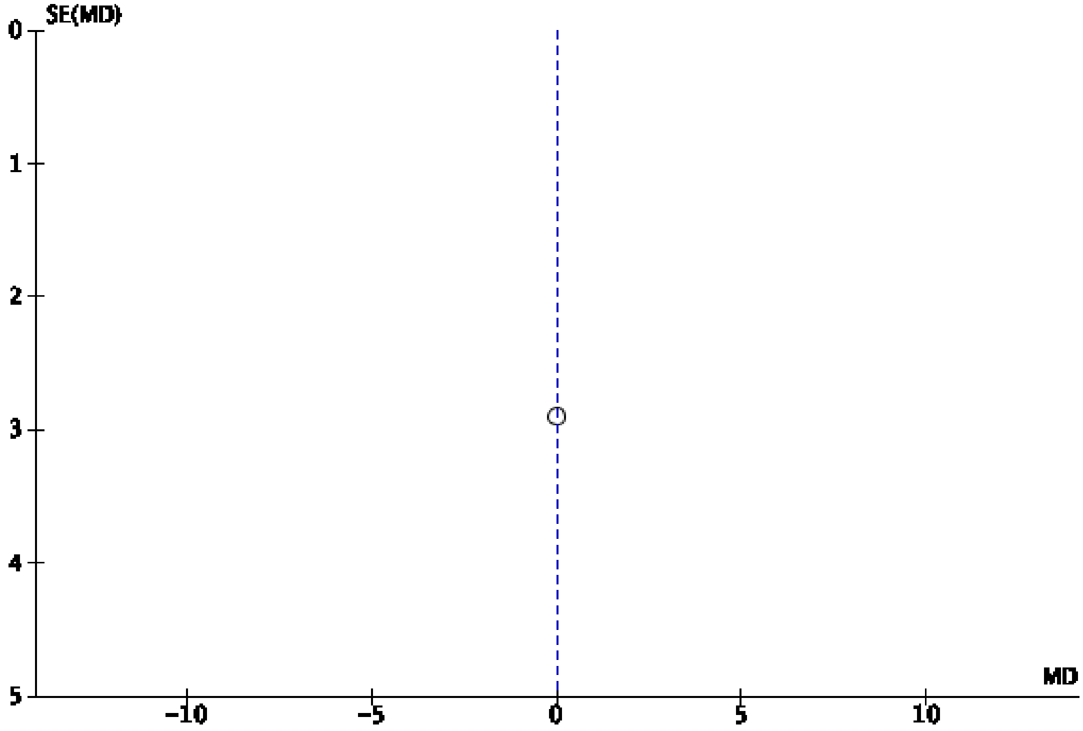

Figure S22. Funnel plot of comparison: SF-36 physical function.

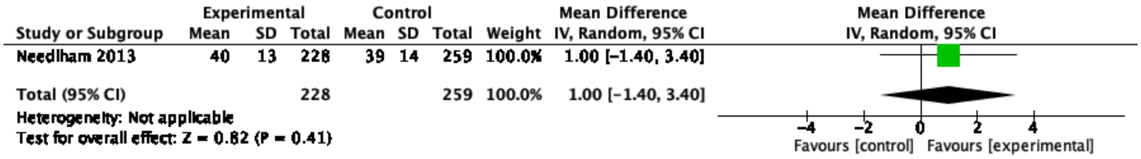

Figure S23. Forest plot of comparison: SF-36 Physical health summary.

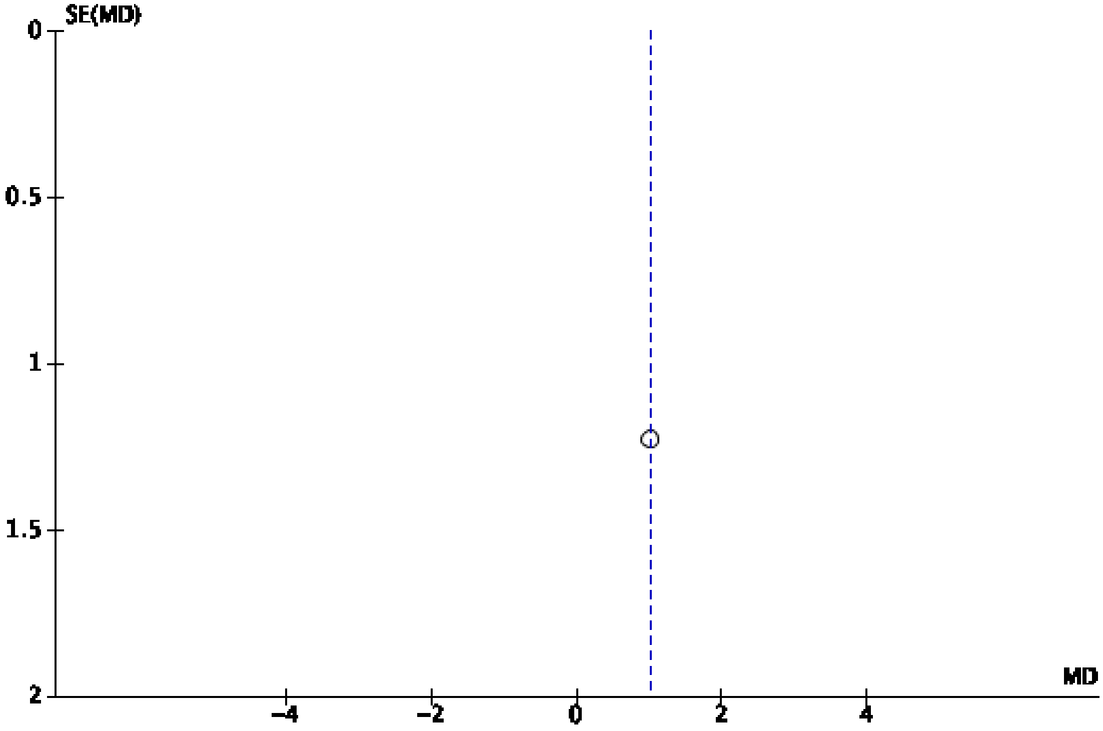

Figure S24. Funnel plot of comparison: SF-36 Physical health summary.

	Experimental			Control				Mean Difference
Study or Subgroup	Mean	SD	Total	Mean	SD	Total	Weight	IV, Random, 95% CI
Needlham 2013	63	26	228	67	25	259	100.0%	−4.00 [−8.55, 0.55]
Total (95% CI)			228			259	100.0%	−4.00 [−8.55, 0.55]

Heterogeneity: Not applicable
Test for overall effect: Z = 1.72 (P = 0.08)

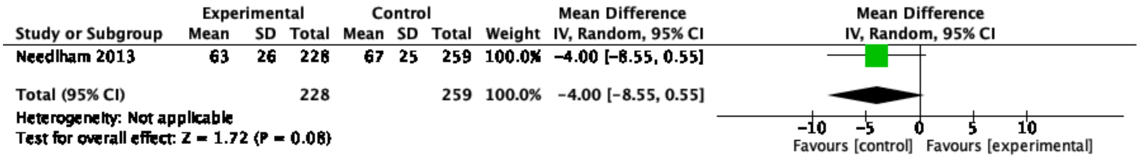

Figure S25. Forest plot of comparison: SF-36 mental.

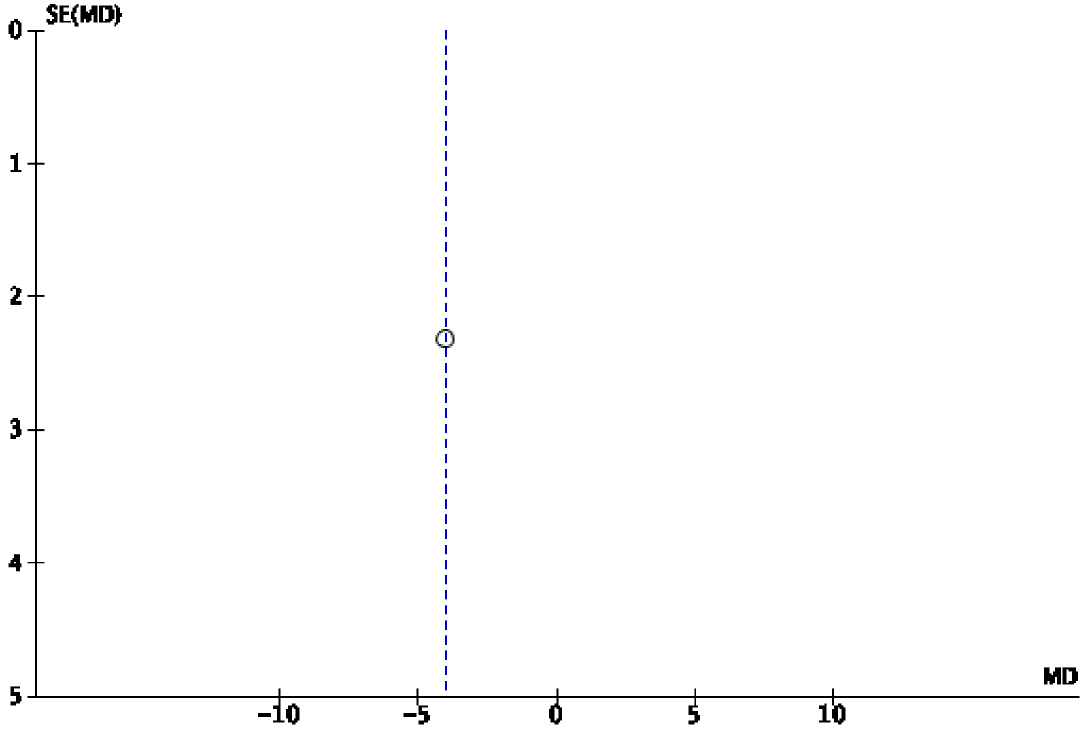

Figure S26. Funnel plot of comparison: SF-36 mental.

Study or Subgroup	Experimental Mean	Experimental SD	Experimental Total	Control Mean	Control SD	Control Total	Weight	Mean Difference IV, Random, 95% CI
Needlham 2013	43	15	228	46	15	259	100.0%	−3.00 [−5.67, −0.33]
Total (95% CI)			228			259	100.0%	−3.00 [−5.67, −0.33]

Heterogeneity: Not applicable
Test for overall effect: Z = 2.20 (P = 0.03)

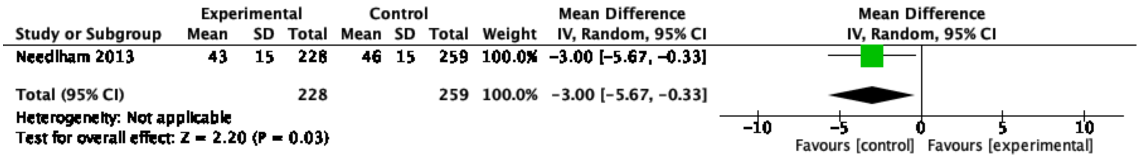

Figure S27. Forest plot of comparison: SF-36 mental health summary.

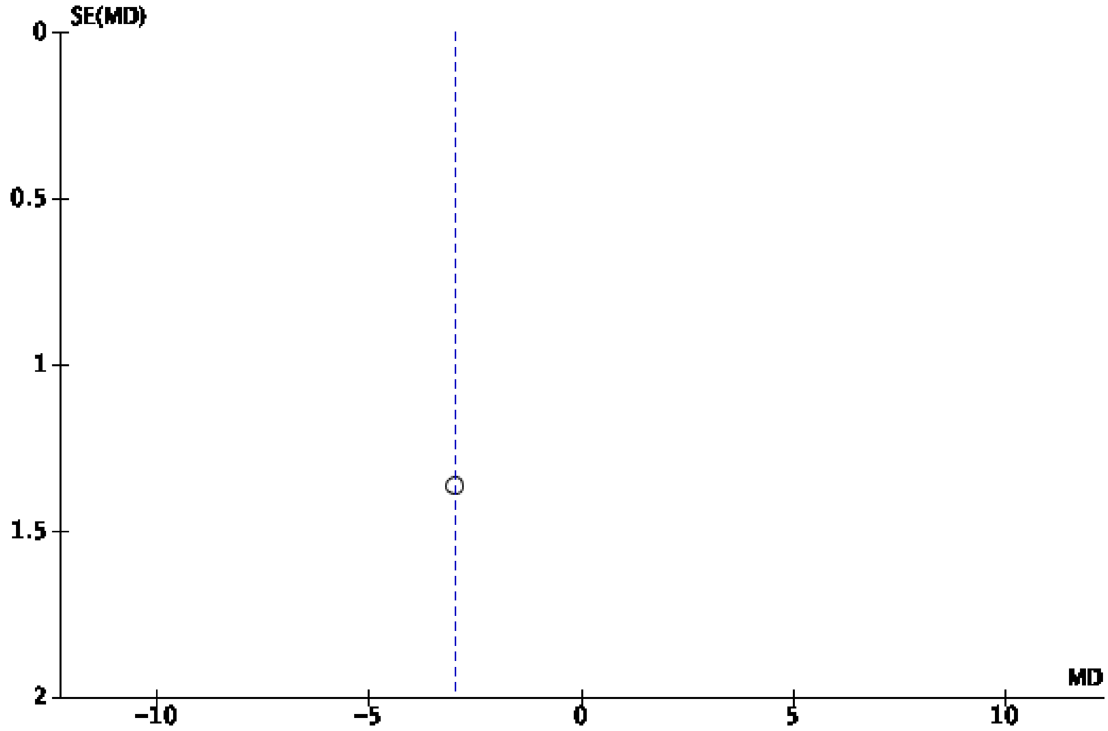

Figure S28. Funnel plot of comparison: SF-36 mental health summary.

Energy: Adverse events

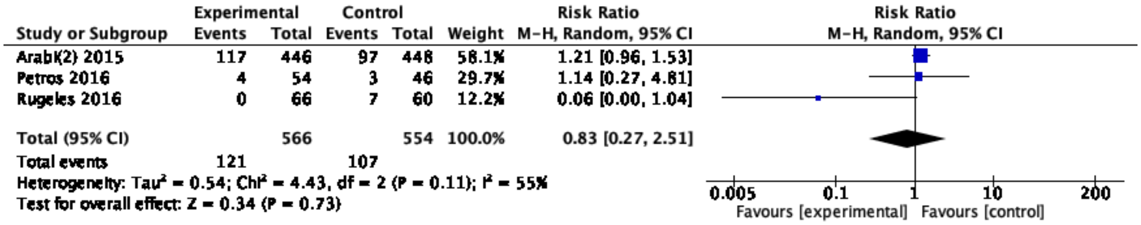

Figure S29. Forest plot of comparison: Diarrhea.

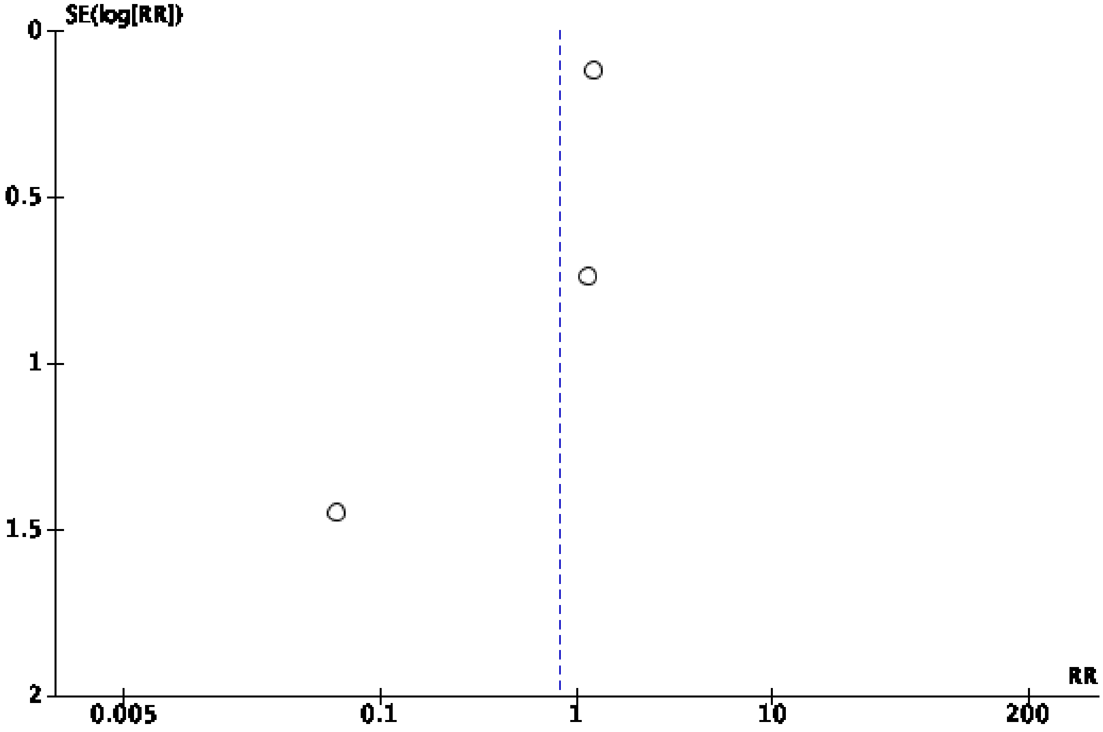

Figure S30. Funnel plot of comparison: Diarrhea.

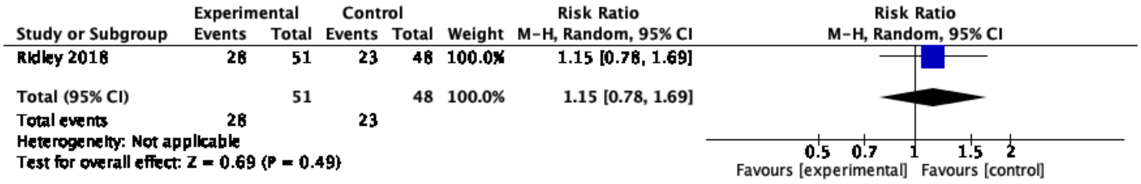

Figure S31. Forest plot of comparison: Residual volume in stomach > 300 ml.

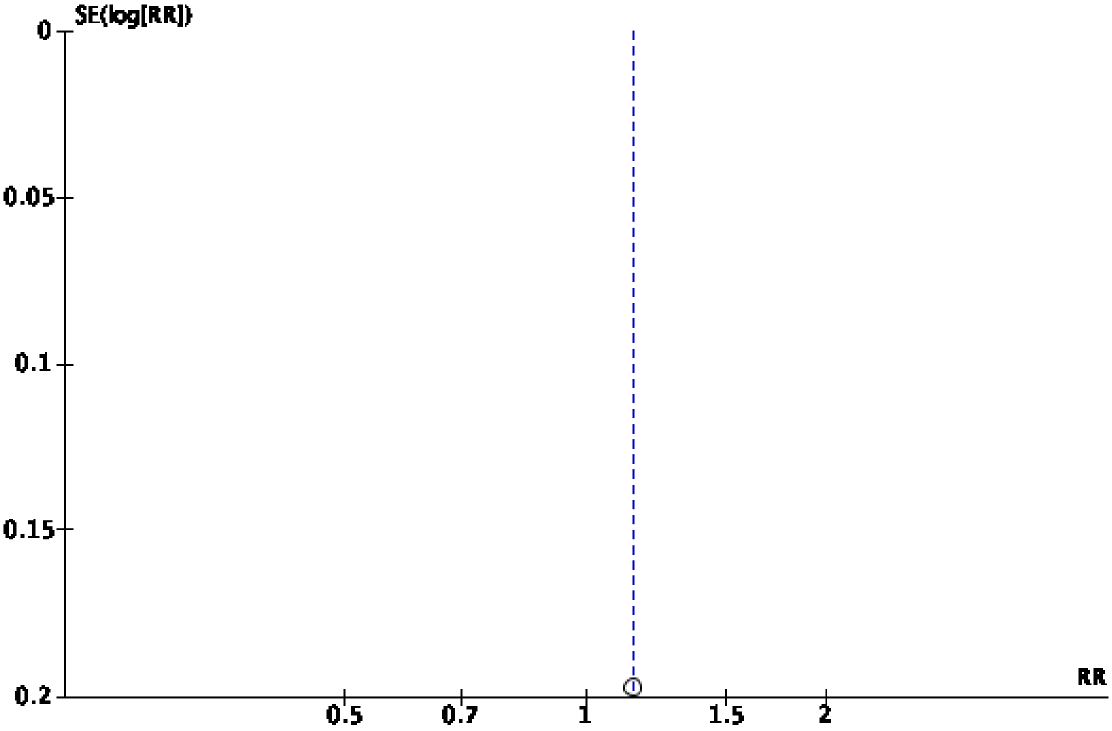

Figure S32. Funnel plot of comparison: Residual volume in stomach > 300 ml.

Study or Subgroup	Experimental Events	Experimental Total	Control Events	Control Total	Weight	Risk Ratio M-H, Random, 95% CI
Ridley 2018	16	51	14	48	100.0%	1.08 [0.59, 1.96]
Total (95% CI)		51		48	100.0%	1.08 [0.59, 1.96]
Total events	16		14			

Heterogeneity: Not applicable
Test for overall effect: Z = 0.24 (P = 0.81)

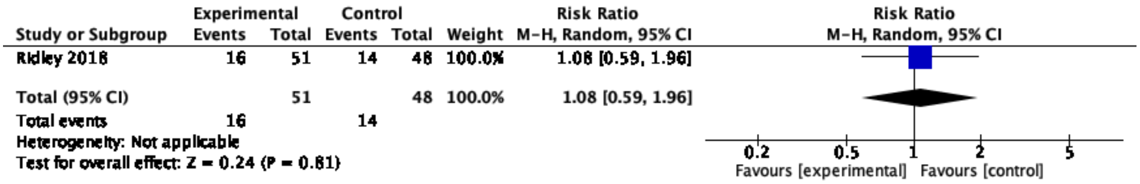

Figure S33. Forest plot of comparison: Abdominal distention.

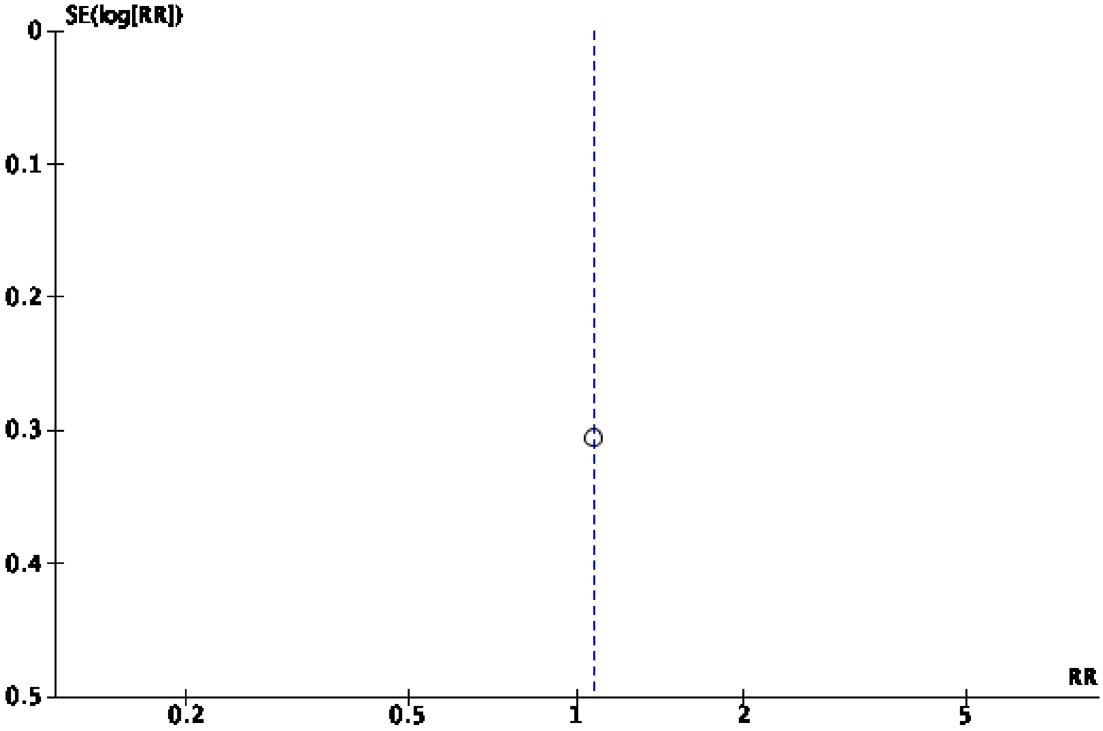

Figure S34. Funnel plot of comparison: Abdominal distention.

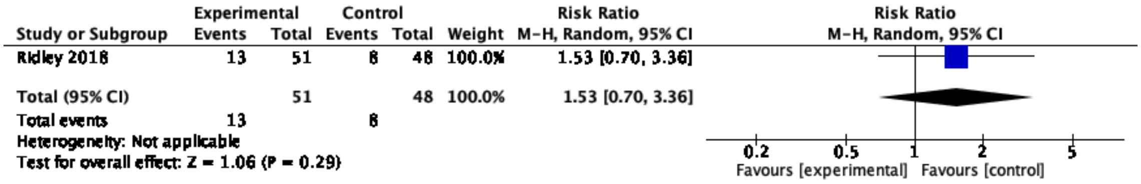

Figure S35. Forest plot of comparison: Vomiting.

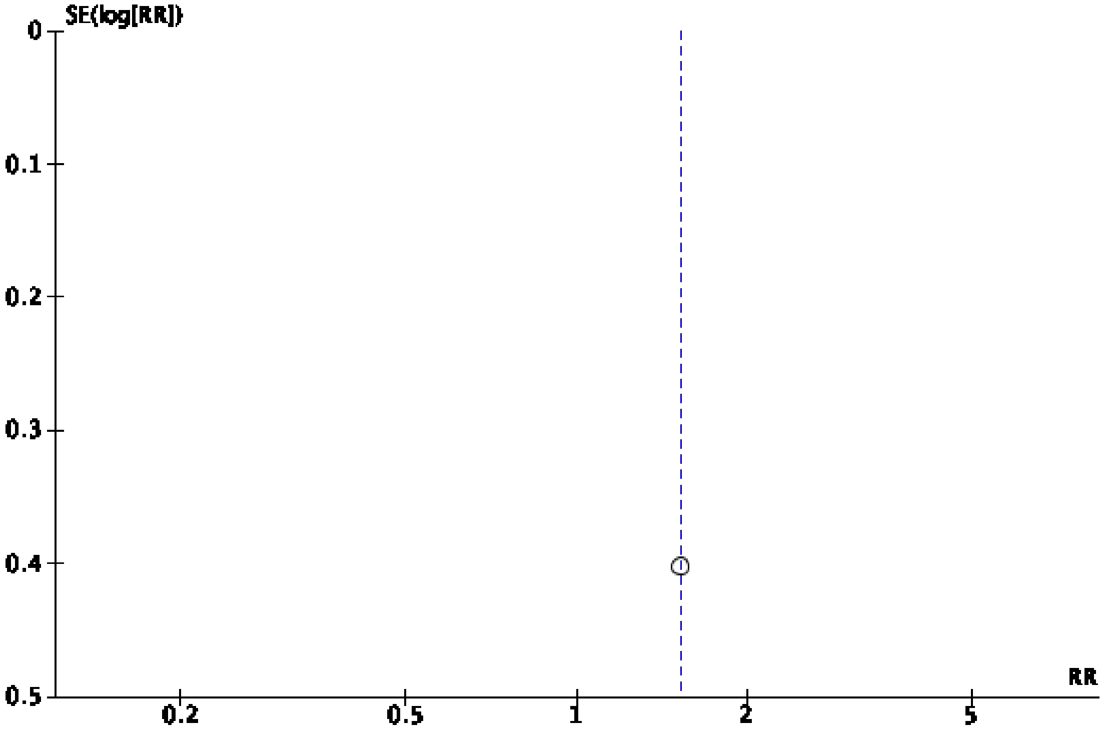

Figure S36. Funnel plot of comparison: Vomiting.

Study or Subgroup	Experimental Events	Experimental Total	Control Events	Control Total	Weight	Risk Ratio M-H, Random, 95% CI	Year
Braunschweig 2015	30	40	26	38	51.9%	1.10 [0.83, 1.45]	2015
Allingstrup 2017	52	100	25	99	48.1%	2.06 [1.40, 3.03]	2017
Total (95% CI)		140		137	100.0%	1.48 [0.75, 2.93]	
Total events	82		51				

Heterogeneity: $Tau^2 = 0.21$; $Chi^2 = 8.09$, df = 1 (P = 0.004); $I^2 = 88\%$
Test for overall effect: Z = 1.14 (P = 0.26)

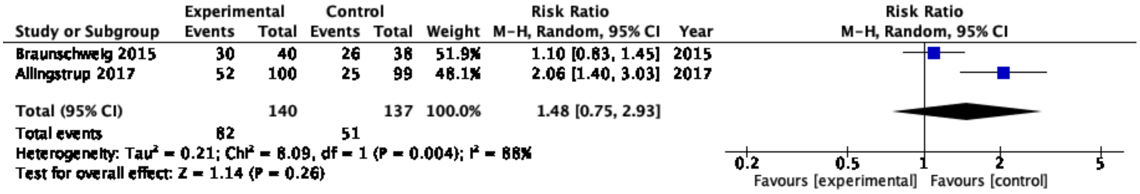

Figure S37. Forest plot of comparison: Hyperglycemia.

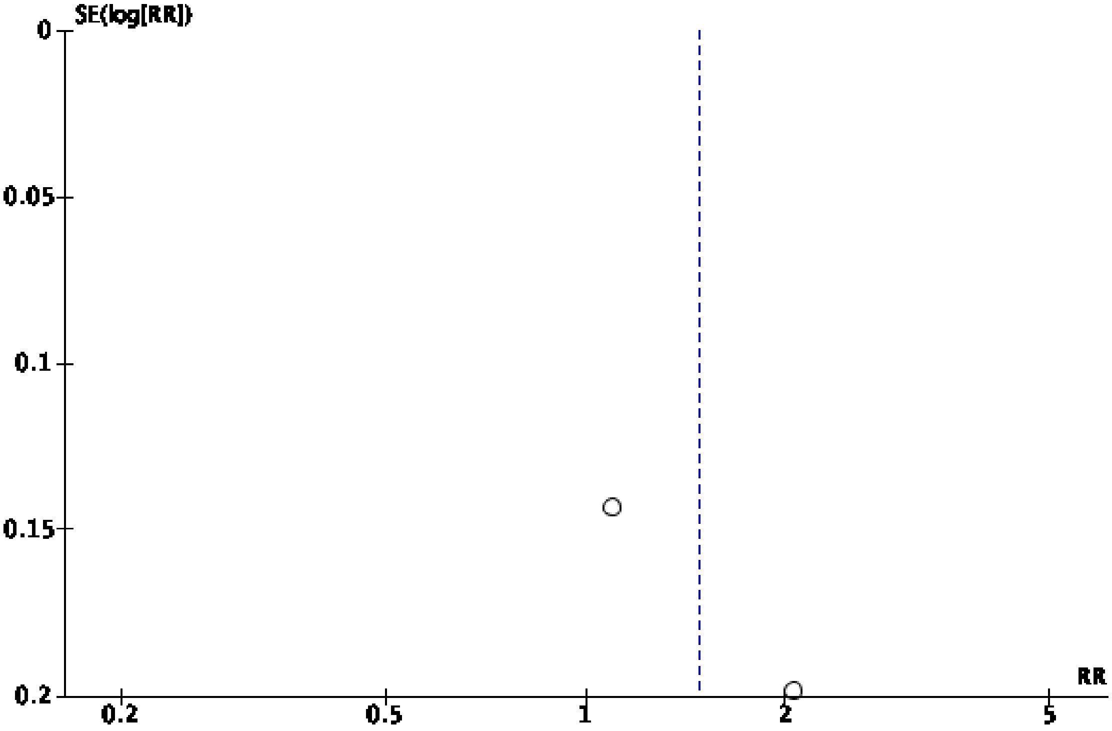

Figure S38. Funnel plot of comparison: Hyperglycemia.

Study or Subgroup	Experimental Events	Experimental Total	Control Events	Control Total	Weight	Risk Ratio M-H, Random, 95% CI	Year
Arabi 2011	21	120	25	120	43.3%	0.84 [0.50, 1.42]	2011
Arabi(2) 2015	7	446	6	448	21.3%	1.17 [0.40, 3.46]	2015
Braunschweig 2015	16	40	6	38	29.4%	2.53 [1.11, 5.79]	2015
Allingstrup 2017	2	100	1	99	6.0%	1.98 [0.18, 21.49]	2017
Total (95% CI)		706		705	100.0%	1.31 [0.71, 2.43]	
Total events	46		38				

Heterogeneity: $Tau^2 = 0.16$; $Chi^2 = 5.10$, df = 3 (P = 0.16); $I^2 = 41\%$
Test for overall effect: Z = 0.87 (P = 0.38)

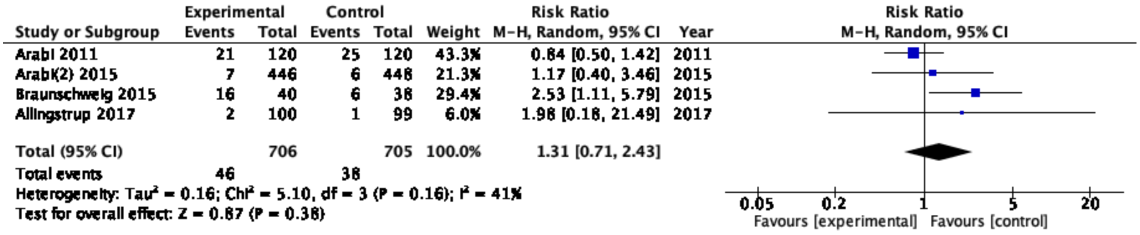

Figure S39. Forest plot of comparison: Hypoglycemia.

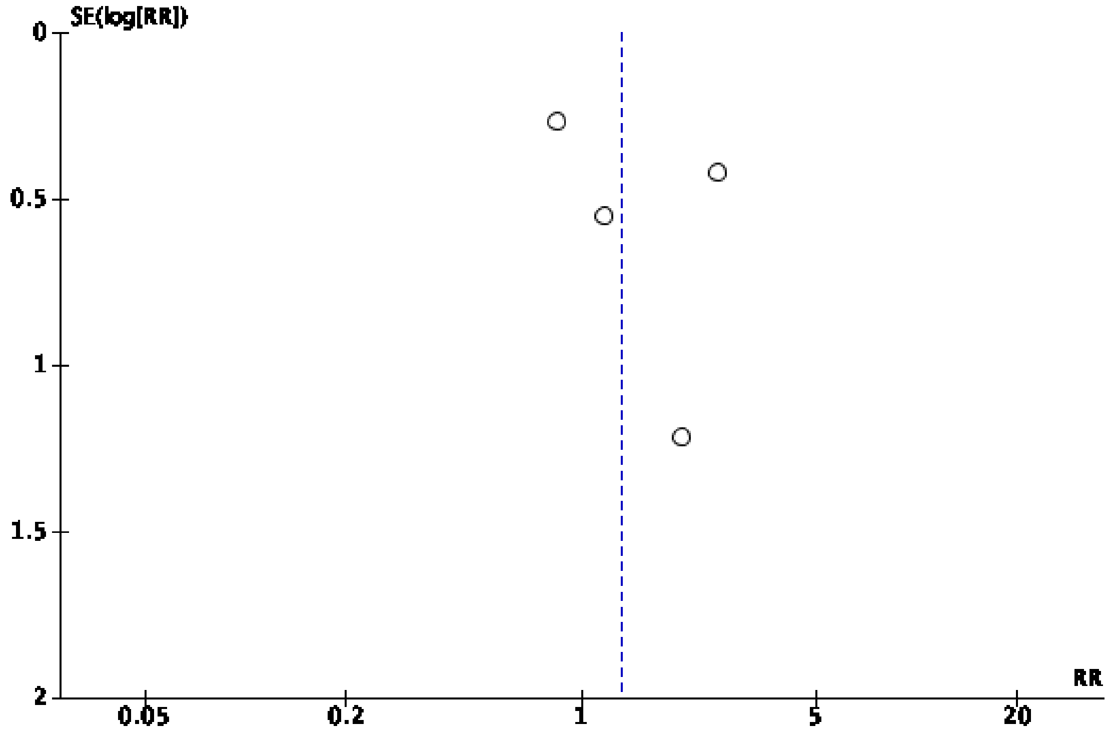

Figure S40. Funnel plot of comparison: Hypoglycemia.

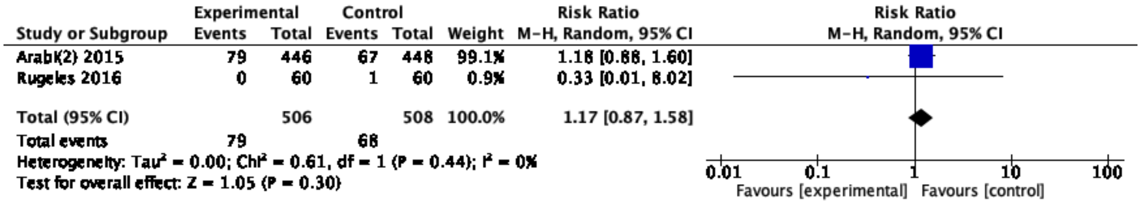

Figure S41. Forest plot of comparison: Gastrointestinal intolerance.

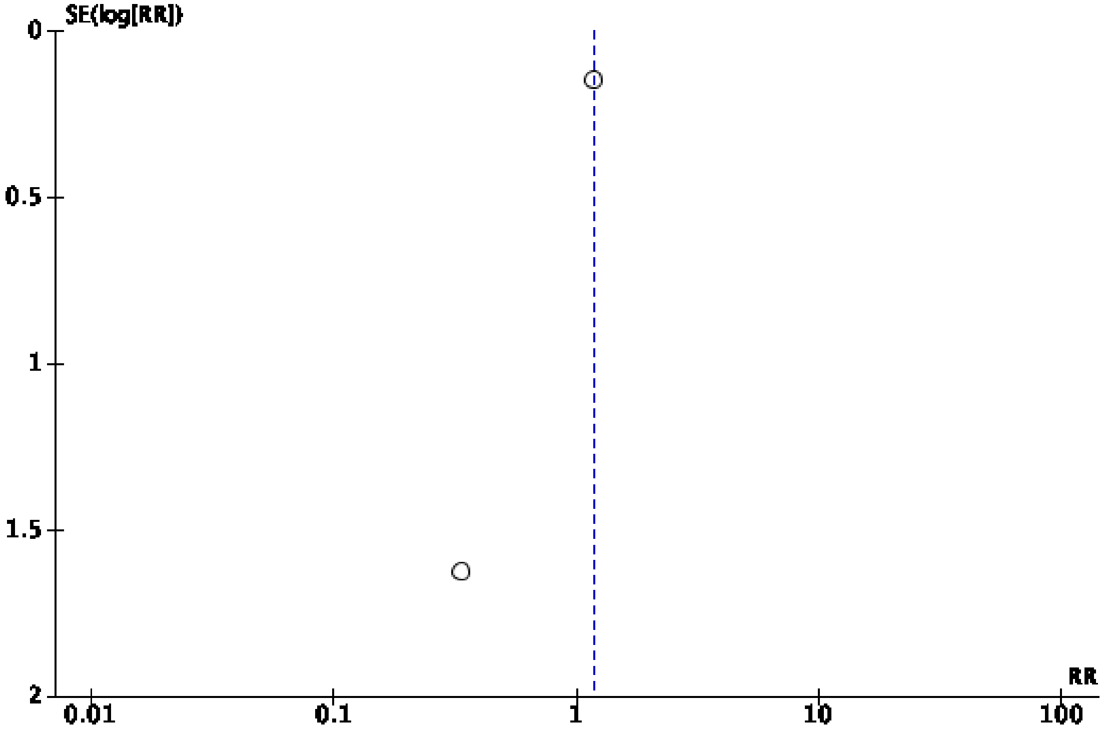

Figure S42. Funnel plot of comparison: Gastrointestinal intolerance.

<u>**Energy: Mortality**</u>

Study or Subgroup	Experimental Events	Experimental Total	Control Events	Control Total	Weight	Risk Ratio M-H, Random, 95% CI	Year
Arabi 2011	28	120	22	120	6.7%	1.27 [0.77, 2.09]	2011
Rice(3) 2012	109	492	118	508	31.8%	0.95 [0.76, 1.20]	2012
Arabi(2) 2015	127	440	121	445	37.4%	1.06 [0.86, 1.31]	2015
Petros 2016	18	54	18	48	6.1%	0.89 [0.53, 1.50]	2016
Rugeles 2016	16	60	18	60	5.1%	0.89 [0.50, 1.57]	2016
Allingstrup 2017	30	100	34	99	10.2%	0.87 [0.58, 1.31]	2017
Wang 2020	11	74	7	76	2.1%	1.61 [0.66, 3.94]	2020
Mousavian 2020	2	29	3	29	0.6%	0.67 [0.12, 3.70]	2020
Total (95% CI)		1369		1385	100.0%	1.00 [0.88, 1.14]	
Total events	341		341				

Heterogeneity: Tau² = 0.00; Chi² = 3.48, df = 7 (P = 0.84); I² = 0%
Test for overall effect: Z = 0.07 (P = 0.95)

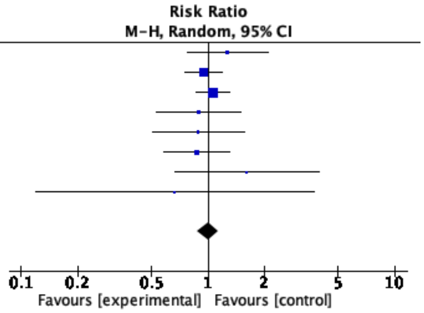

Figure S43. Forest plot of comparison: Mortality.

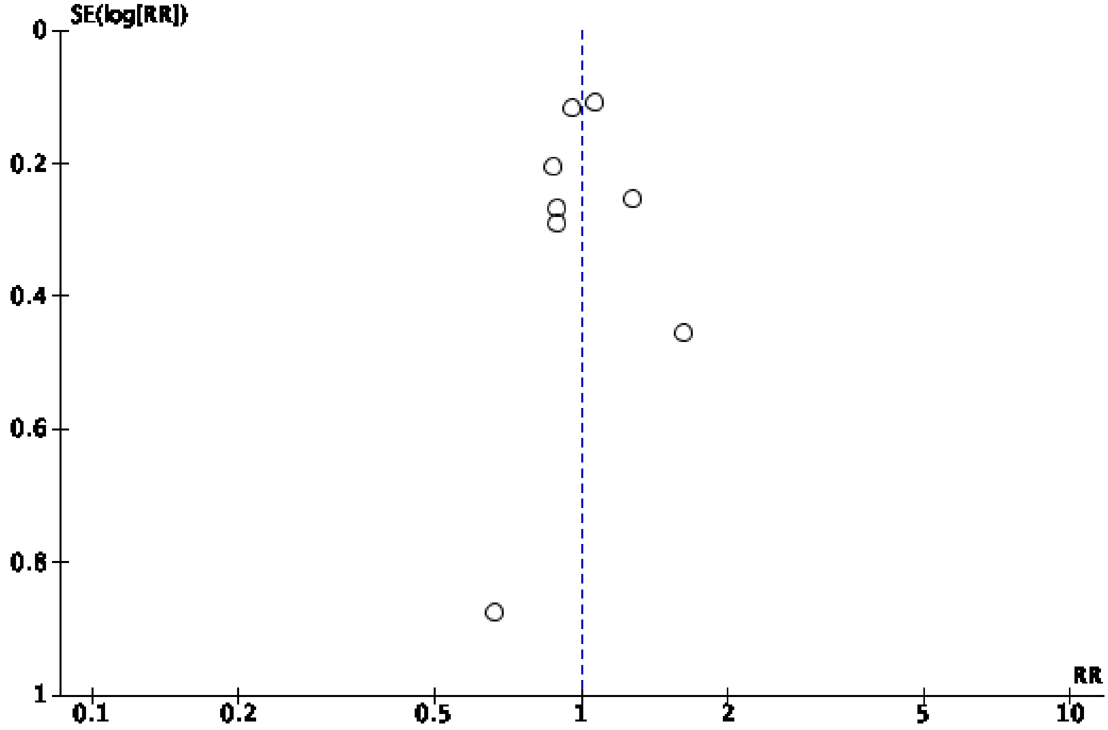

Figure S44. Funnel plot of comparison: Mortality.

<u>**Energy: Length of hospital stay**</u>

Study or Subgroup	Experimental Mean	Experimental SD	Experimental Total	Control Mean	Control SD	Control Total	Weight	Mean Difference IV, Random, 95% CI	Year
Arabi 2011	67.2	93.6	120	70.2	106.9	120	2.1%	−3.00 [−28.42, 22.42]	2011
Charles 2014	31	2.5	42	35.2	4.9	41	38.9%	−4.20 [−5.88, −2.52]	2014
Braunschweig 2015	27.2	18.2	40	22.8	14.3	38	16.6%	4.40 [−2.84, 11.64]	2015
Allingstrup 2017	30	30.37	100	34	28.89	99	14.1%	−4.00 [−12.24, 4.24]	2017
Ridley 2018	22	21	51	23	17	48	15.9%	−1.00 [−8.51, 6.51]	2018
McKeever 2020	25.3	18.4	19	20.4	7.2	16	12.5%	4.90 [−4.09, 13.89]	2020
Total (95% CI)			372			362	100.0%	−1.08 [−4.86, 2.70]	

Heterogeneity: $Tau^2 = 8.83$; $Chi^2 = 9.01$, df = 5 (P = 0.11); $I^2 = 45\%$

Test for overall effect: Z = 0.56 (P = 0.58)

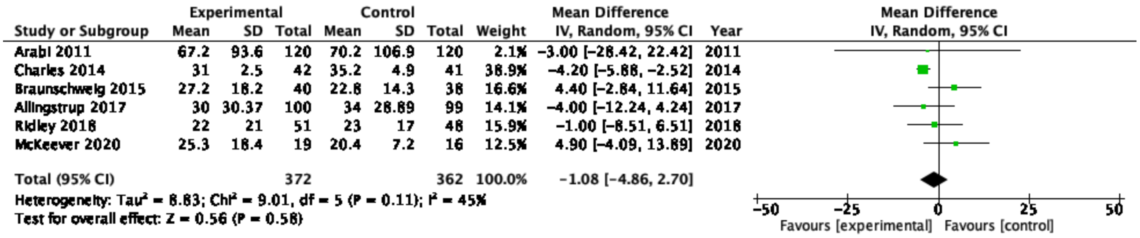

Figure S45. Forest plot of comparison: Length of hospital stay.

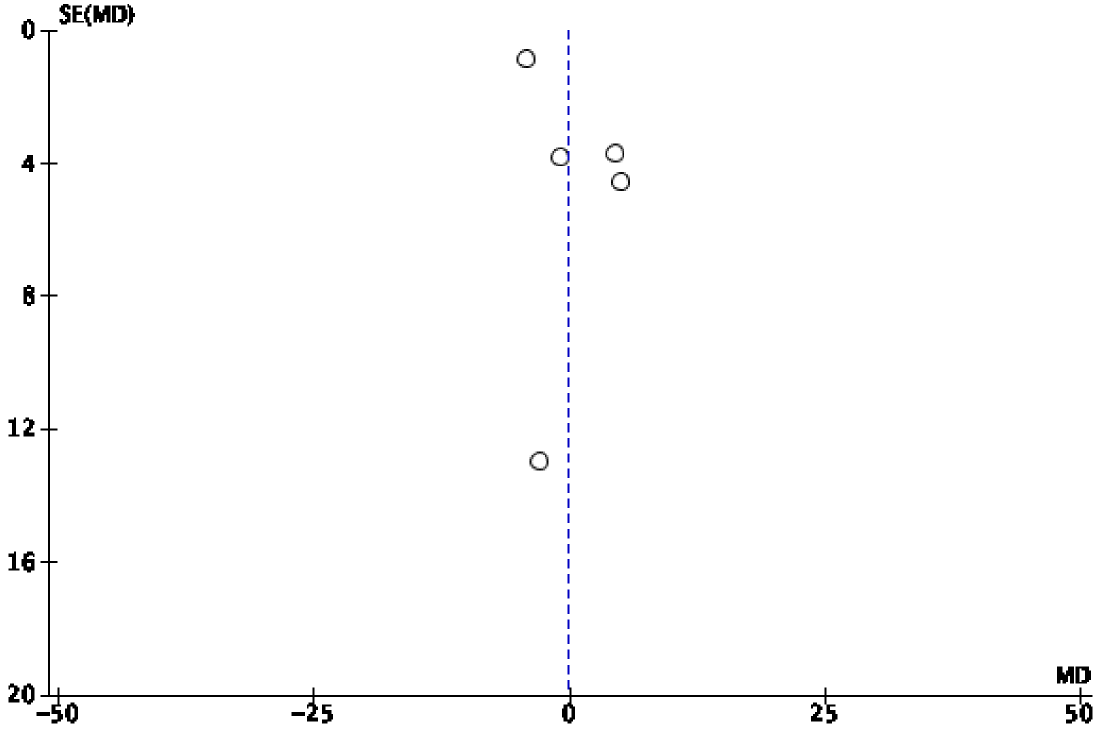

Figure S46. Funnel plot of comparison: Length of hospital stay.

3. Risk of bias and effect on outcomes about optimal protein delivery

Protein: Risk of bias summary and graph

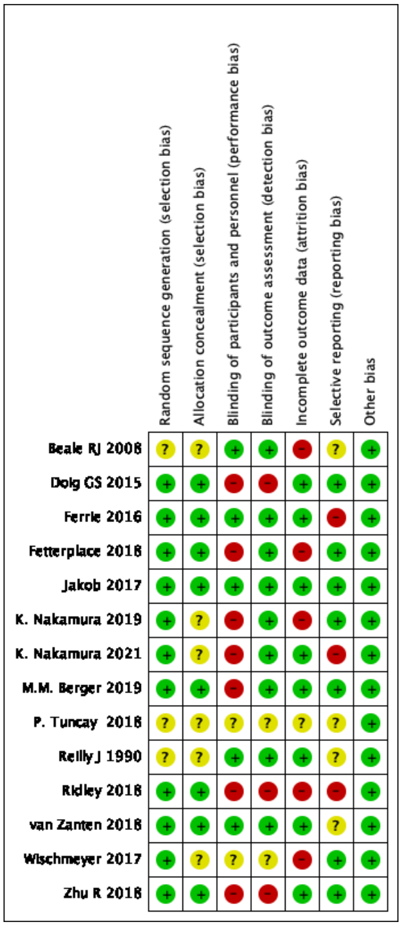

Figure S47. Risk of bias summary: review authors' judgements about each risk of bias item for each included study.

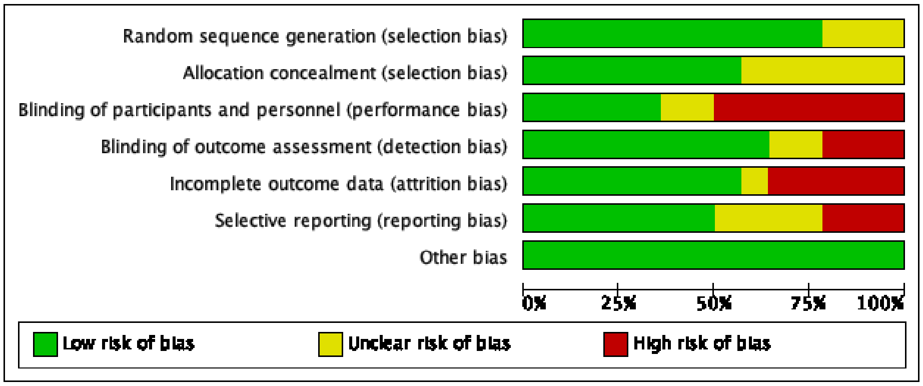

Figure S48. Risk of bias graph: review authors' judgements about each risk of bias item presented as percentages across all included studies.

Protein: ADL at the hospital discharge

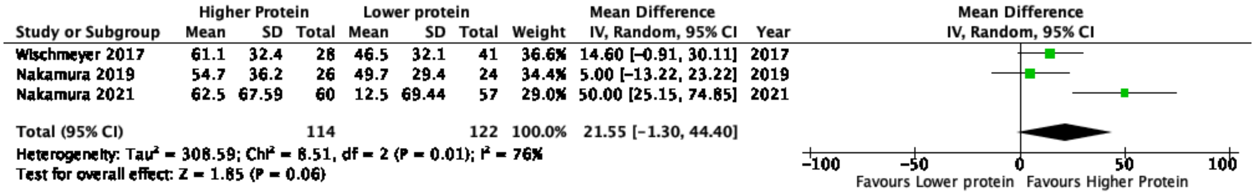

Figure S49. Forest plot of comparison: Barthel Index.

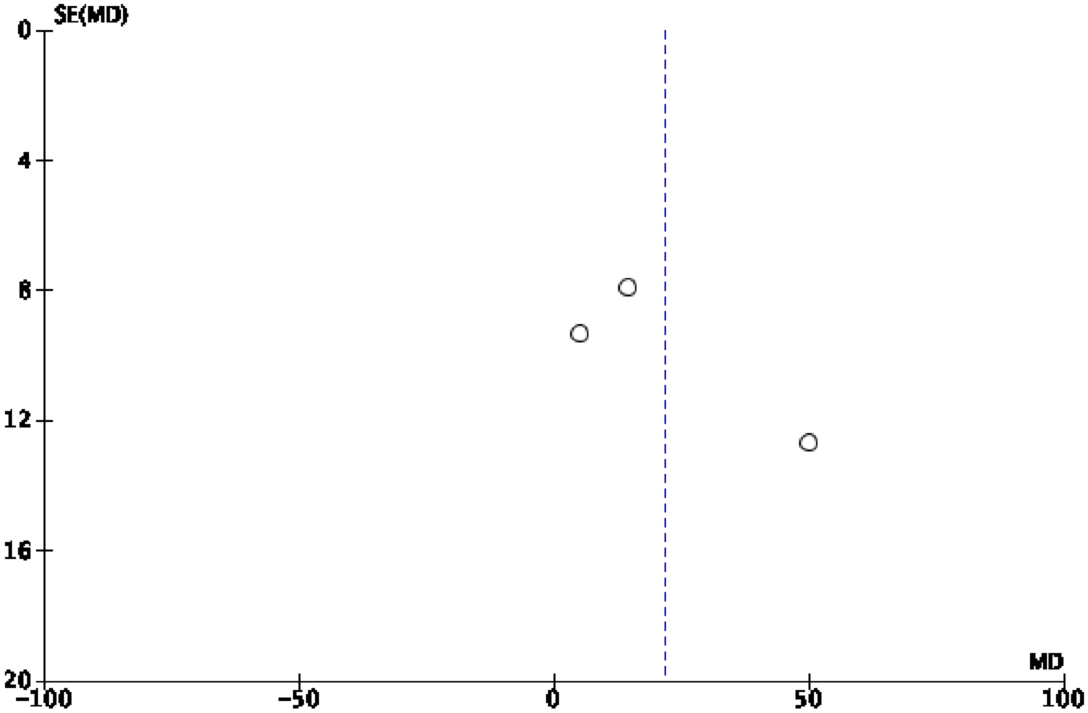

Figure S50. Funnel plot of comparison: Barthel Index.

Protein: Physical functions at the hospital discharge or one year after the hospital discharge

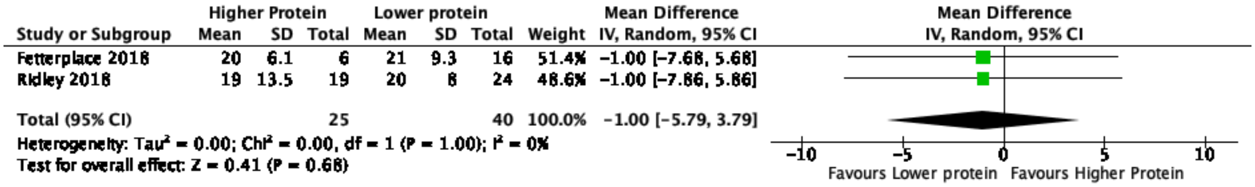

Figure S51. Forest plot of comparison: Handgrip strength.

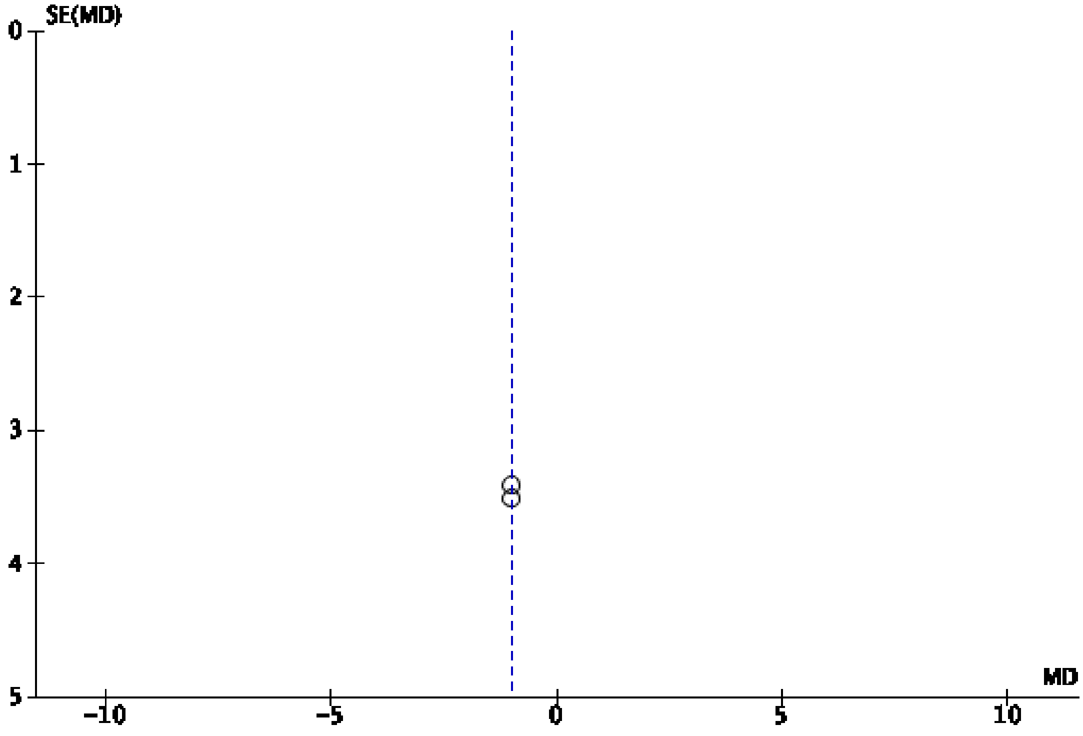

Figure S52. Funnel plot of comparison: Handgrip strength.

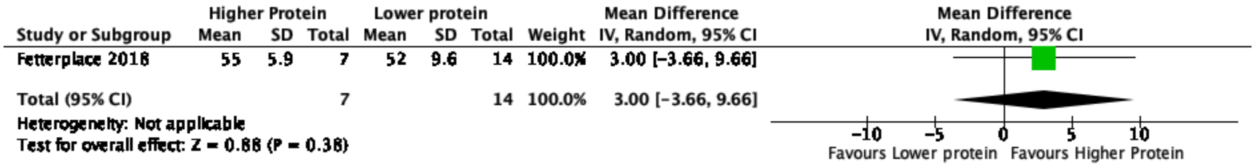

Figure S53. Forest plot of comparison: MRC-sum score.

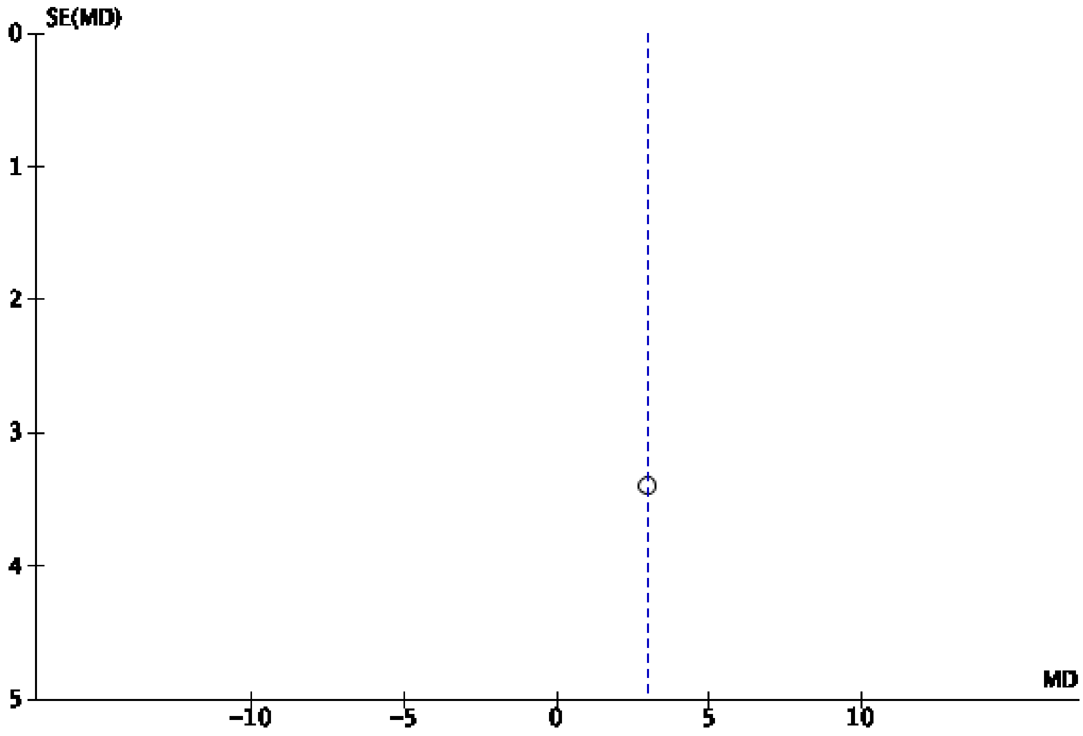

Figure S54. Funnel plot of comparison: MRC-sum score.

Protein: Muscle mass change during hospital stay

Evaluation using ultrasound or computed tomography

Study or Subgroup	Higher Protein Mean	SD	Total	Lower protein Mean	SD	Total	Weight	Std. Mean Difference IV, Random, 95% CI	Year
Ferrie 2016	8.4	1	59	7.9	1.1	60	41.6%	0.47 [0.11, 0.84]	2016
Nakamura 2019	−12.9	8.5	60	−16.9	7	57	40.7%	0.51 [0.14, 0.88]	2019
Nakamura 2021	−11.4	8.1	26	−14.4	7.1	24	17.6%	0.39 [−0.17, 0.95]	2021
Total (95% CI)			145			141	100.0%	0.47 [0.24, 0.71]	

Heterogeneity: $Tau^2 = 0.00$; $Chi^2 = 0.13$, df = 2 ($P = 0.94$); $I^2 = 0\%$
Test for overall effect: Z = 3.93 ($P < 0.0001$)

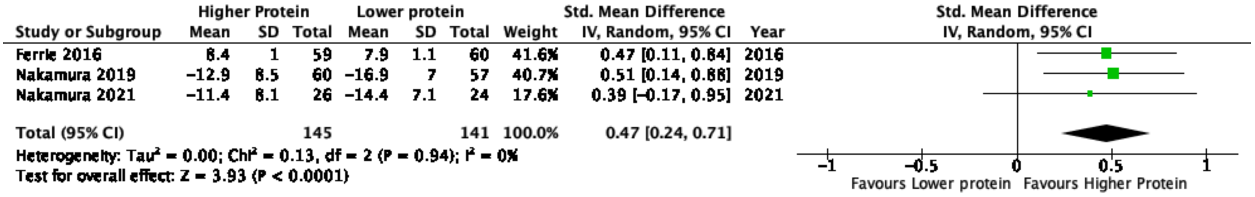

Figure S55. Forest plot of comparison: Muscle mass change evaluated by ultrasound or computed tomography.

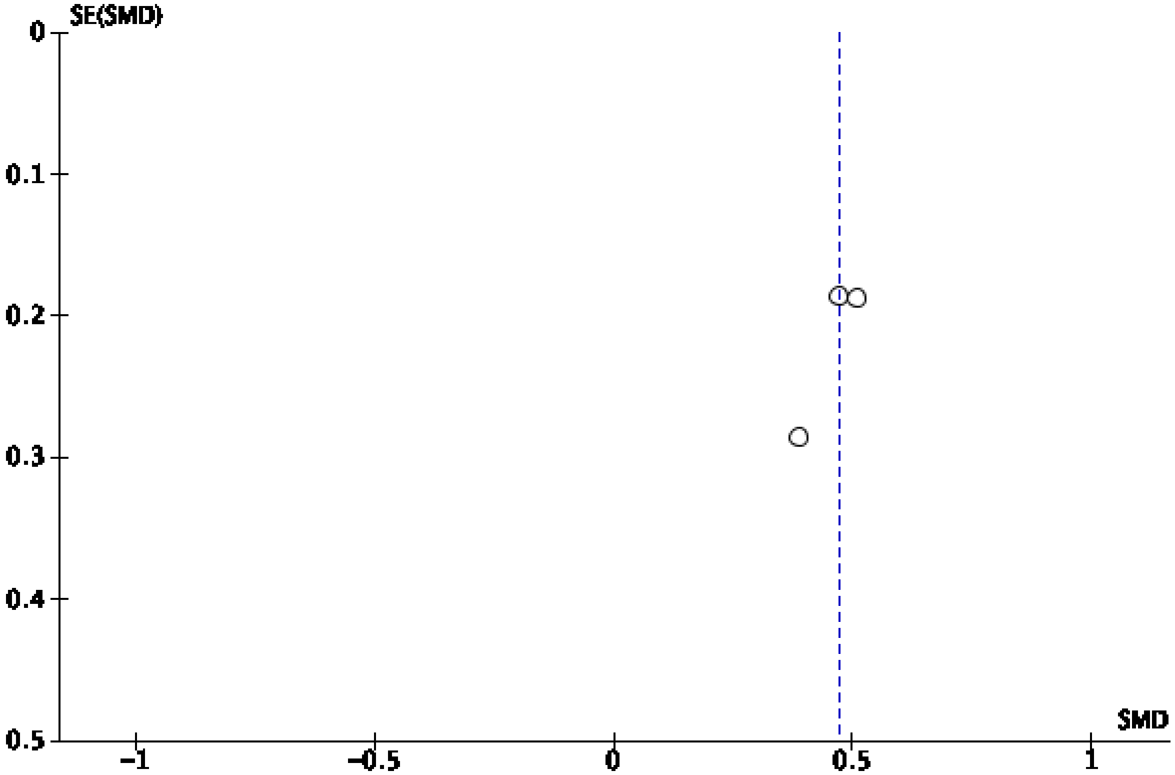

Figure S56. Funnel plot of comparison: Muscle mass change evaluated by ultrasound or computed tomography.

Evaluation using ultrasound only

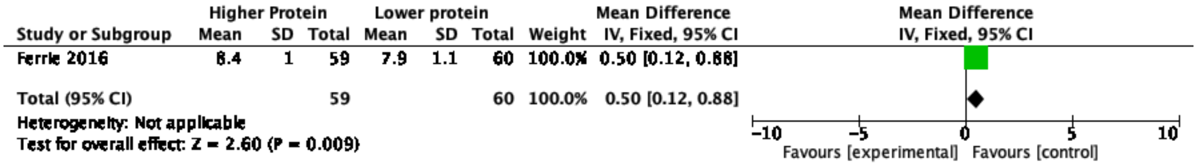

Figure S57. Forest plot of comparison: Muscle mass change evaluated by ultrasound.

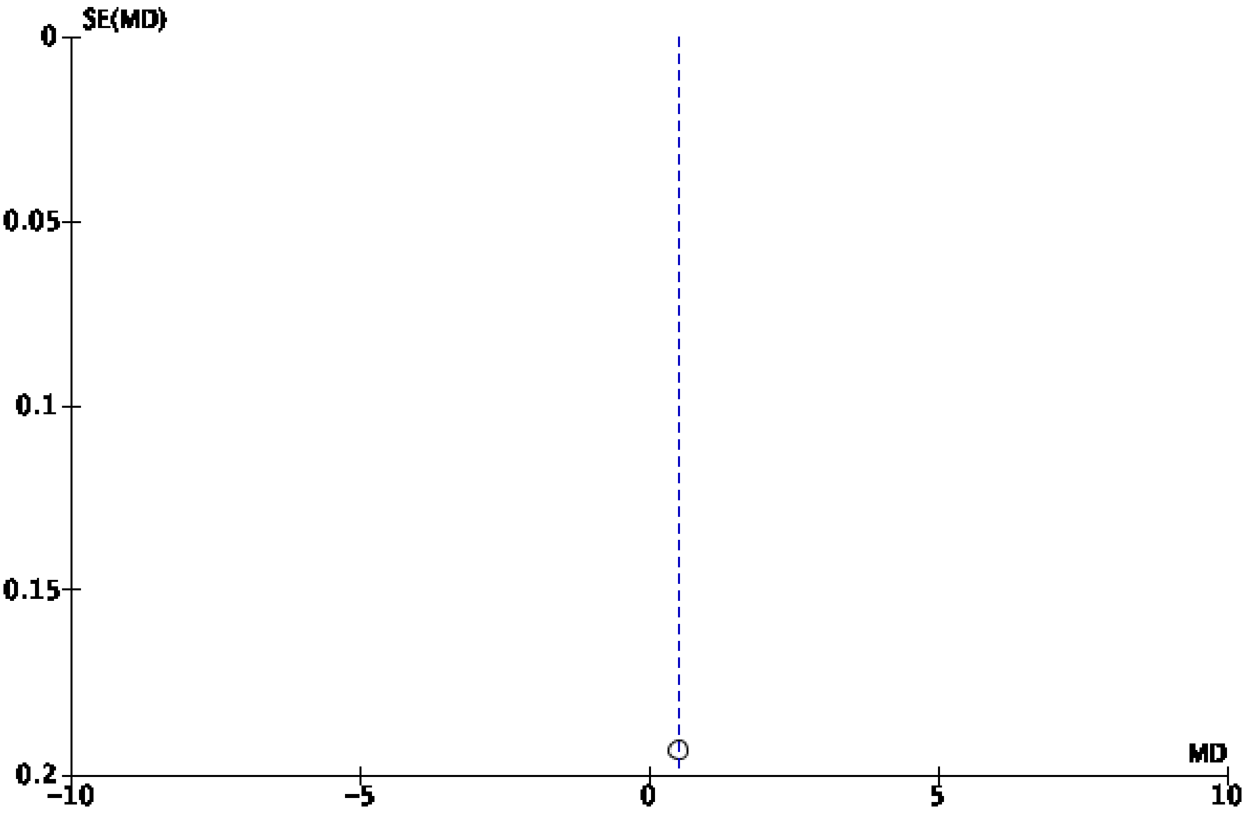

Figure S58. Funnel plot of comparison: Muscle mass change evaluated by ultrasound.

Evaluation using computed tomography only

Study or Subgroup	Higher Protein Mean	Higher Protein SD	Higher Protein Total	Lower protein Mean	Lower protein SD	Lower protein Total	Weight	Mean Difference IV, Fixed, 95% CI
Nakamura 2019	−12.9	8.5	60	−16.9	7	57	69.1%	4.00 [1.18, 6.82]
Nakamura 2021	−11.4	8.1	26	−14.4	7.1	24	30.9%	3.00 [−1.21, 7.21]
Total (95% CI)			86			81	100.0%	3.69 [1.35, 6.03]
Heterogeneity: Chi² = 0.15, df = 1 (P = 0.70); I² = 0%								
Test for overall effect: Z = 3.09 (P = 0.002)								

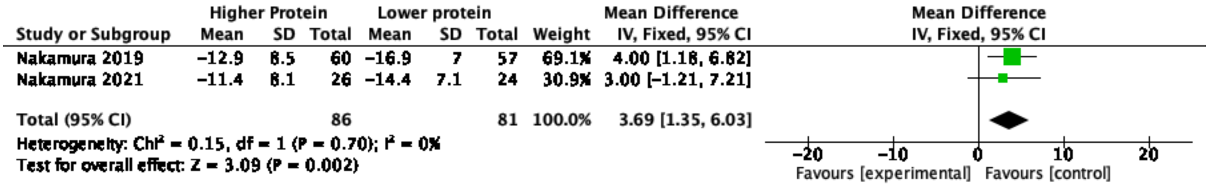

Figure S59. Forest plot of comparison: Muscle mass change evaluated by computed tomography.

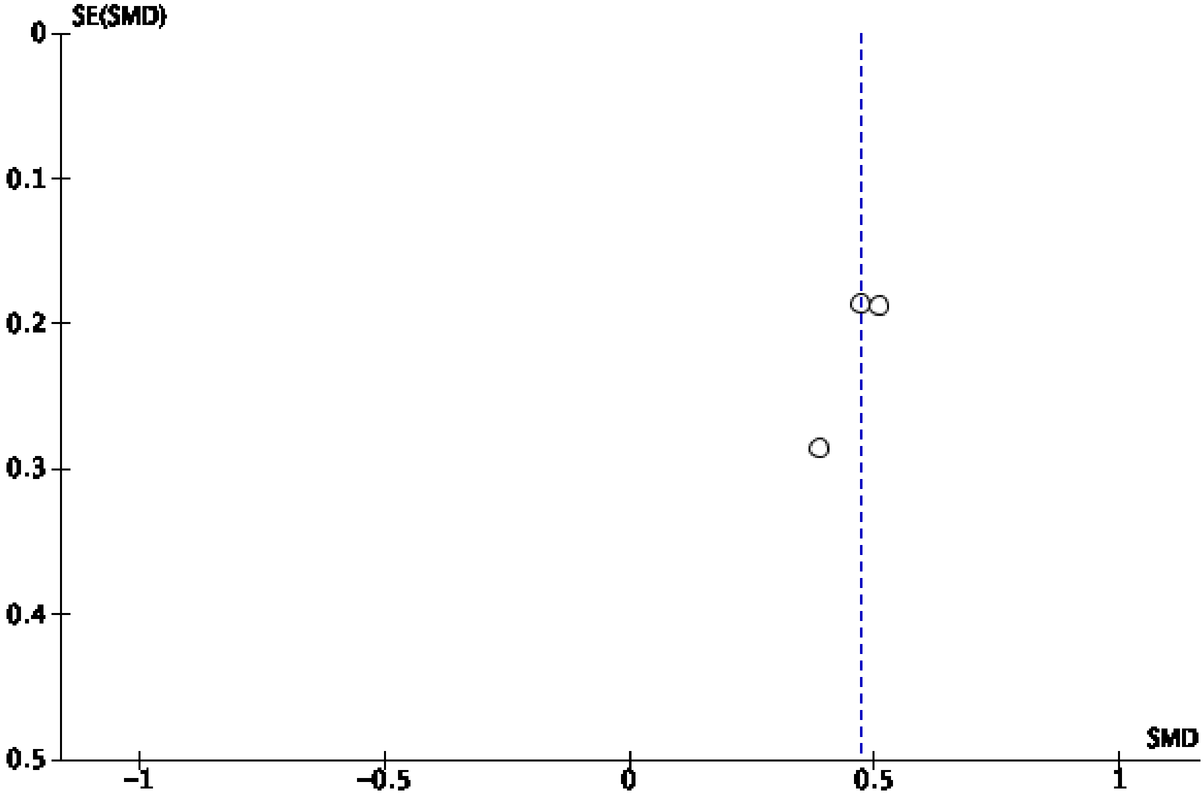

Figure S60. Funnel plot of comparison: Muscle mass change evaluated by computed tomography.

Protein: QOL score at the hospital discharge or one year after the hospital discharge

Study or Subgroup	Higher Protein Mean	Higher Protein SD	Higher Protein Total	Lower protein Mean	Lower protein SD	Lower protein Total	Weight	Std. Mean Difference IV, Random, 95% CI	Year
Doig 2015	50.5	27.7	192	52.8	25.9	180	44.2%	-0.09 [-0.29, 0.12]	2015
Ridley 2018	0.69	0.18	35	0.76	0.17	29	7.4%	-0.39 [-0.89, 0.10]	2018
Zhu 2018	49.5	26.7	149	52.5	32.8	142	34.5%	-0.10 [-0.33, 0.13]	2018
Nakamura 2021	8	7	60	8	6.7	57	13.9%	0.00 [-0.36, 0.36]	2021
Total (95% CI)			436			408	100.0%	-0.10 [-0.24, 0.03]	

Heterogeneity: $Tau^2 = 0.00$; $Chi^2 = 1.65$, df = 3 (P = 0.65); $I^2 = 0\%$
Test for overall effect: Z = 1.47 (P = 0.14)

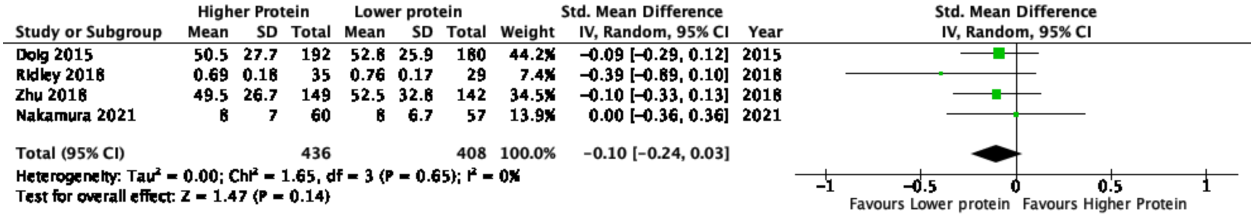

Figure S61. Forest plot of comparison: General (RAND-36 General Health、EQ-5D-5L).

■Funnel plot

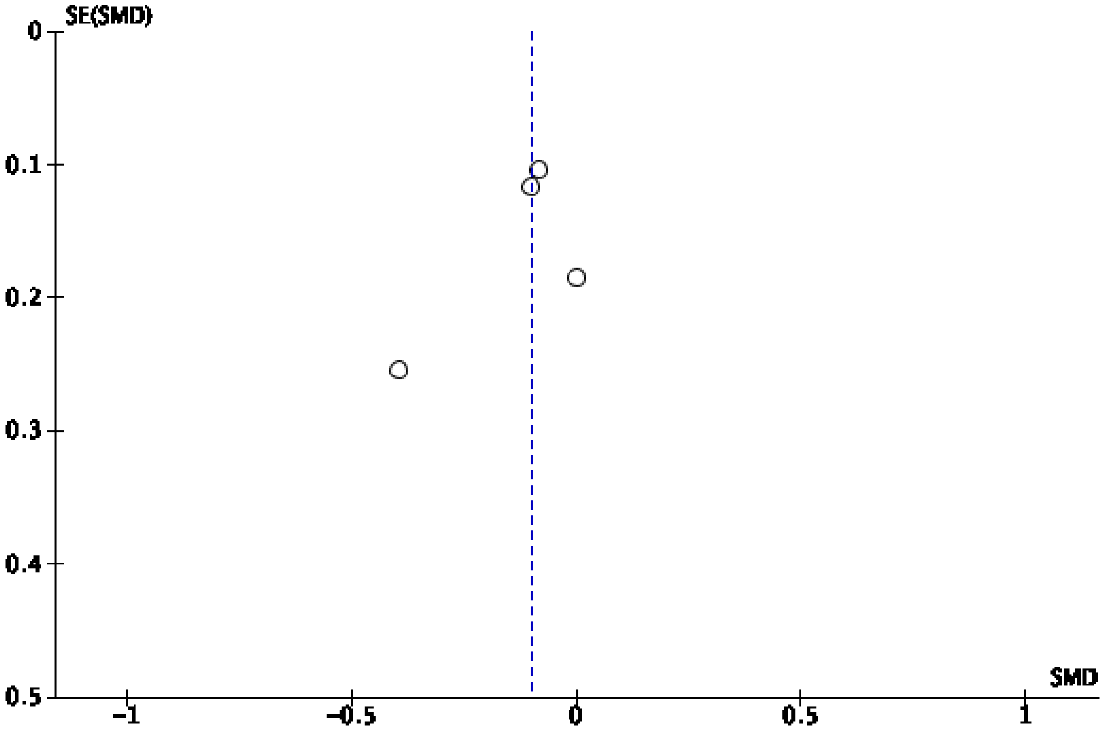

Figure S62. Funnel plot of comparison: General (RAND-36 General Health、EQ5D5L).

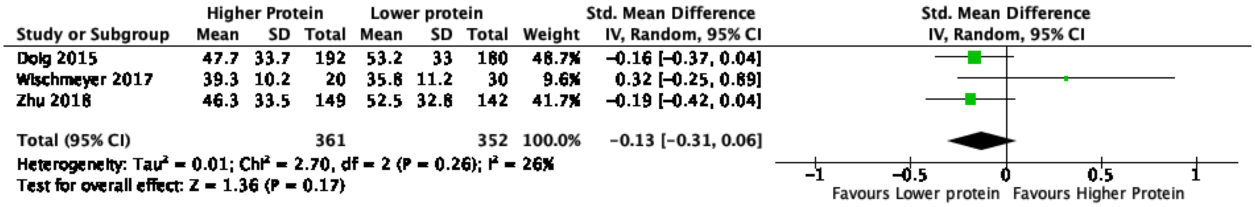

Figure S63. Forest plot of comparison: Physical (RAND-36 physical function, SF-36 physical component).

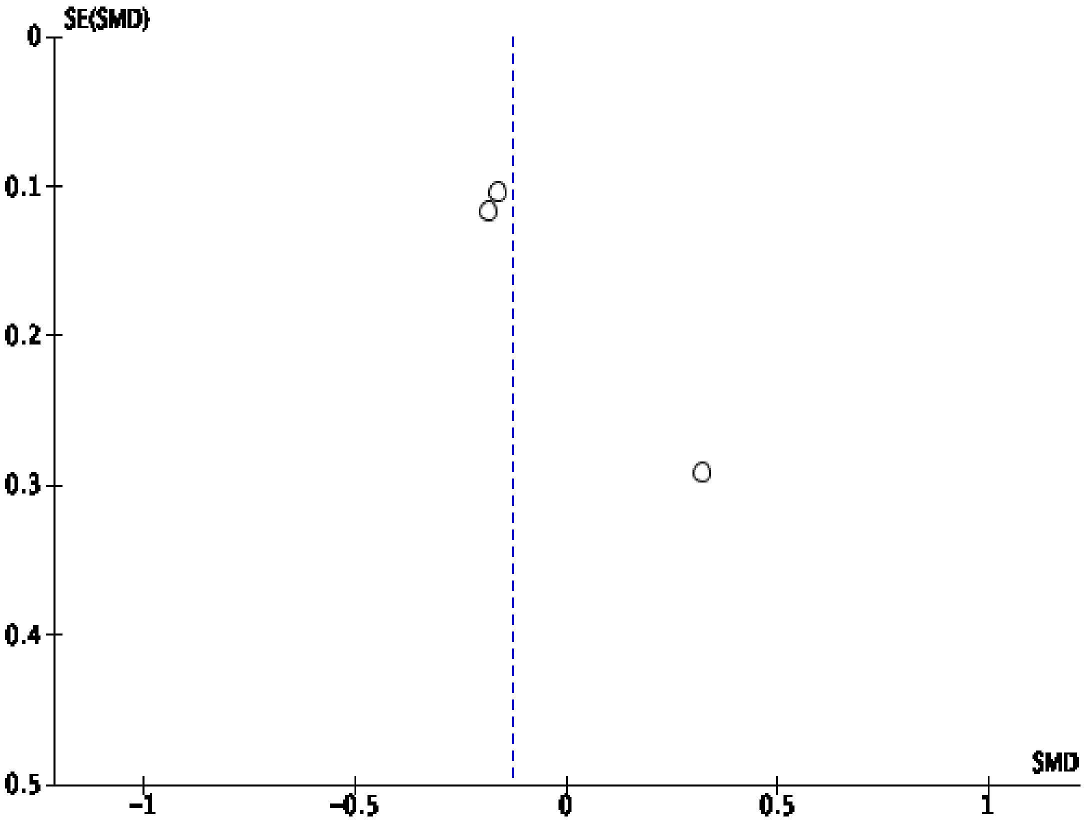

Figure S64. Funnel plot of comparison: Physical (RAND-36 physical function, SF-36 physical component).

Protein: Adverse events

Study or Subgroup	Higher Protein Events	Higher Protein Total	Lower protein Events	Lower protein Total	Weight	Risk Ratio M-H, Random, 95% CI	Year
Beale 2008	21	27	12	28	18.6%	1.81 [1.13, 2.91]	2008
Jakob 2017	29	46	31	44	22.6%	0.89 [0.67, 1.20]	2017
Tuncay 2018	2	23	13	23	6.0%	0.15 [0.04, 0.61]	2018
van Zanten 2018	8	22	11	22	14.0%	0.73 [0.36, 1.45]	2018
Fetterplace 2018	16	30	16	30	18.6%	1.00 [0.62, 1.61]	2018
Nakamura 2019	3	26	2	27	4.2%	1.56 [0.28, 8.58]	2019
Nakamura 2021	14	60	19	57	16.1%	0.70 [0.39, 1.26]	2021
Total (95% CI)		234		231	100.0%	0.90 [0.61, 1.31]	
Total events	93		104				

Heterogeneity: $Tau^2 = 0.15$; $Chi^2 = 16.79$, df = 6 (P = 0.01); $I^2 = 64\%$

Test for overall effect: Z = 0.56 (P = 0.57)

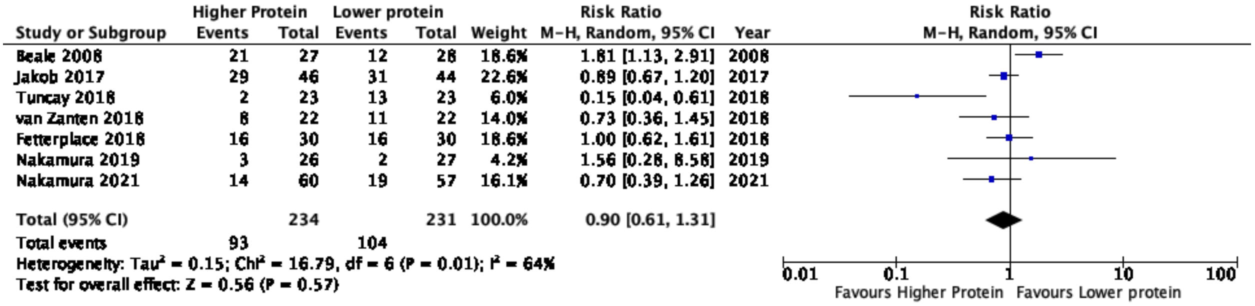

Figure S65. Forest plot of comparison: Diarrhea.

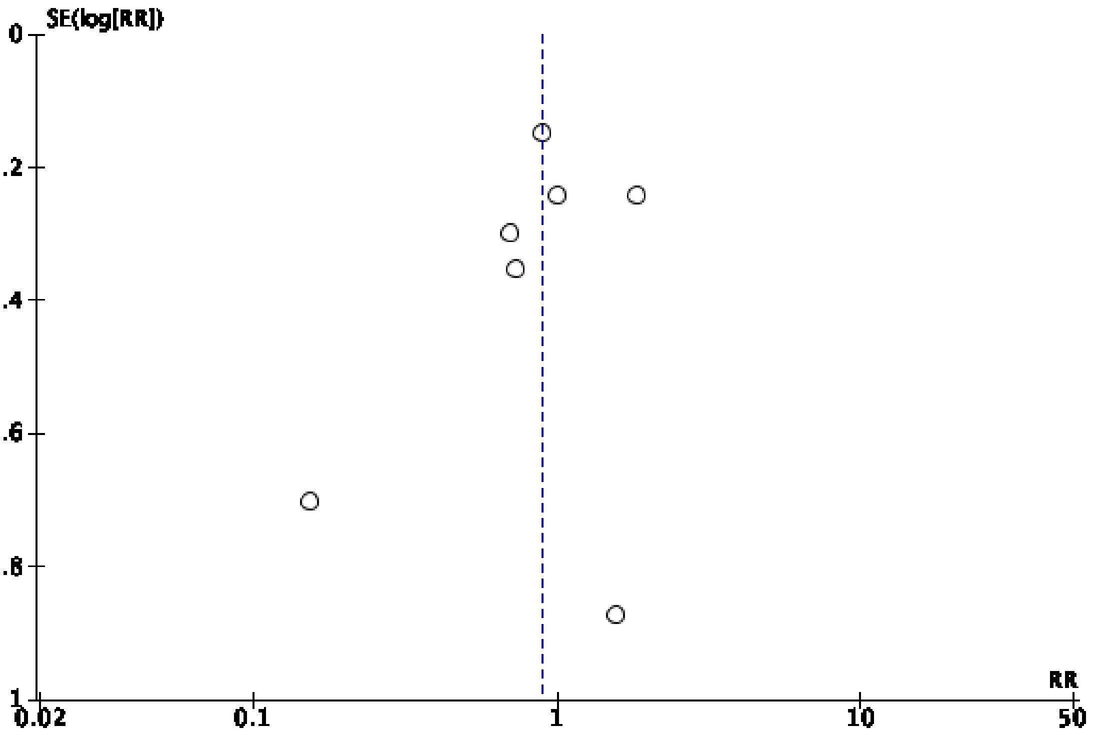

Figure S66. Funnel plot of comparison: Diarrhea.

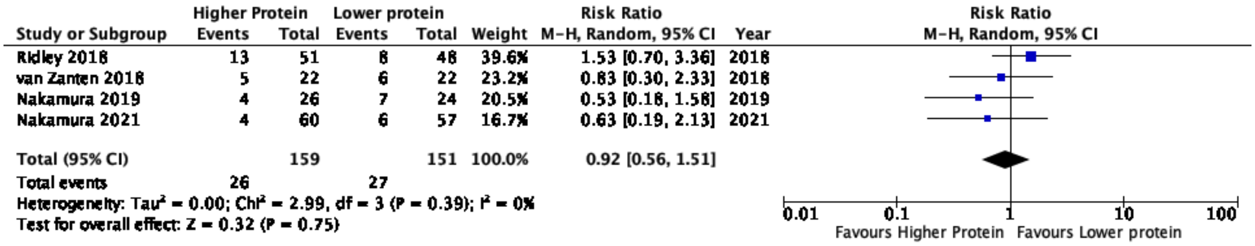

Figure S67. Forest plot of comparison: Vomiting

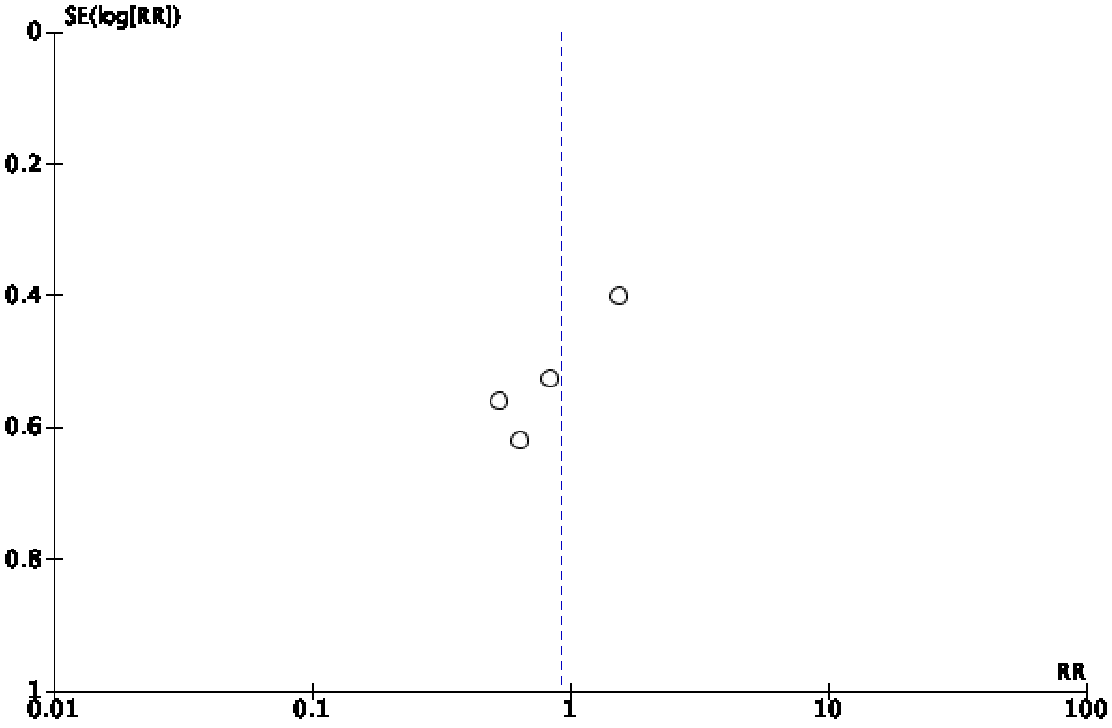

Figure S68. Funnel plot of comparison: Vomiting

Study or Subgroup	Higher Protein Events	Higher Protein Total	Lower protein Events	Lower protein Total	Weight	Risk Ratio M-H, Random, 95% CI	Year
Ridley 2018	28	51	23	48	60.0%	1.15 [0.78, 1.69]	2018
van Zanten 2018	4	22	5	22	13.1%	0.80 [0.25, 2.59]	2018
Fetterplace 2018	9	30	8	30	24.4%	1.13 [0.50, 2.52]	2018
Nakamura 2021	0	60	5	57	2.4%	0.09 [0.00, 1.53]	2021
Total (95% CI)		163		157	100.0%	1.02 [0.65, 1.61]	
Total events	41		41				

Heterogeneity: $Tau^2 = 0.05$; $Chi^2 = 3.75$, $df = 3$ ($P = 0.29$); $I^2 = 20\%$
Test for overall effect: $Z = 0.09$ ($P = 0.93$)

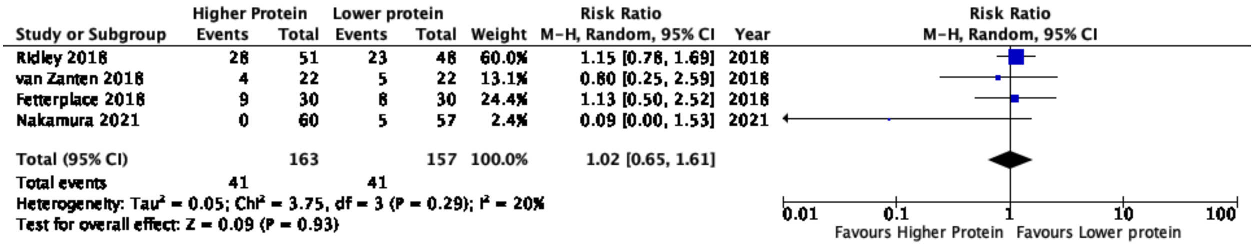

Figure S69. Forest plot of comparison: High gastric residual volume.

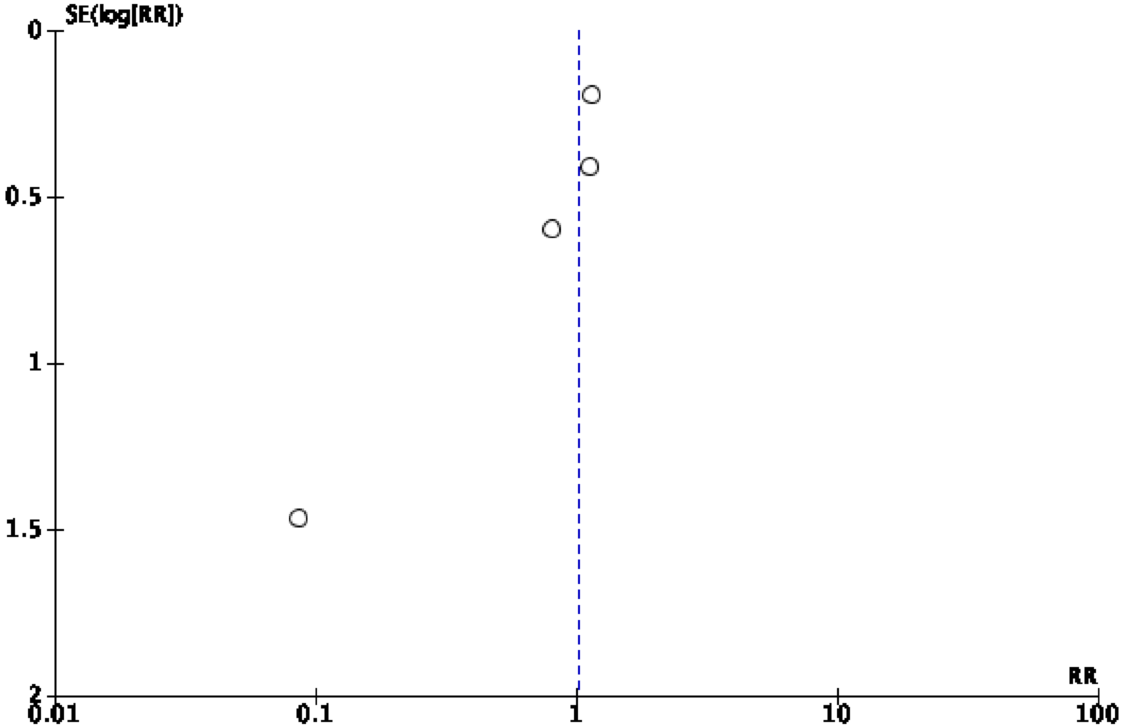

Figure S70. Funnel plot of comparison: High gastric residual volume.

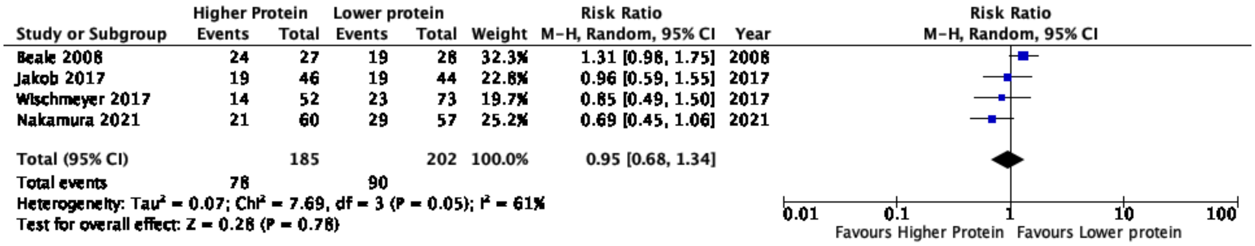

Figure S71. Forest plot of comparison: Infections.

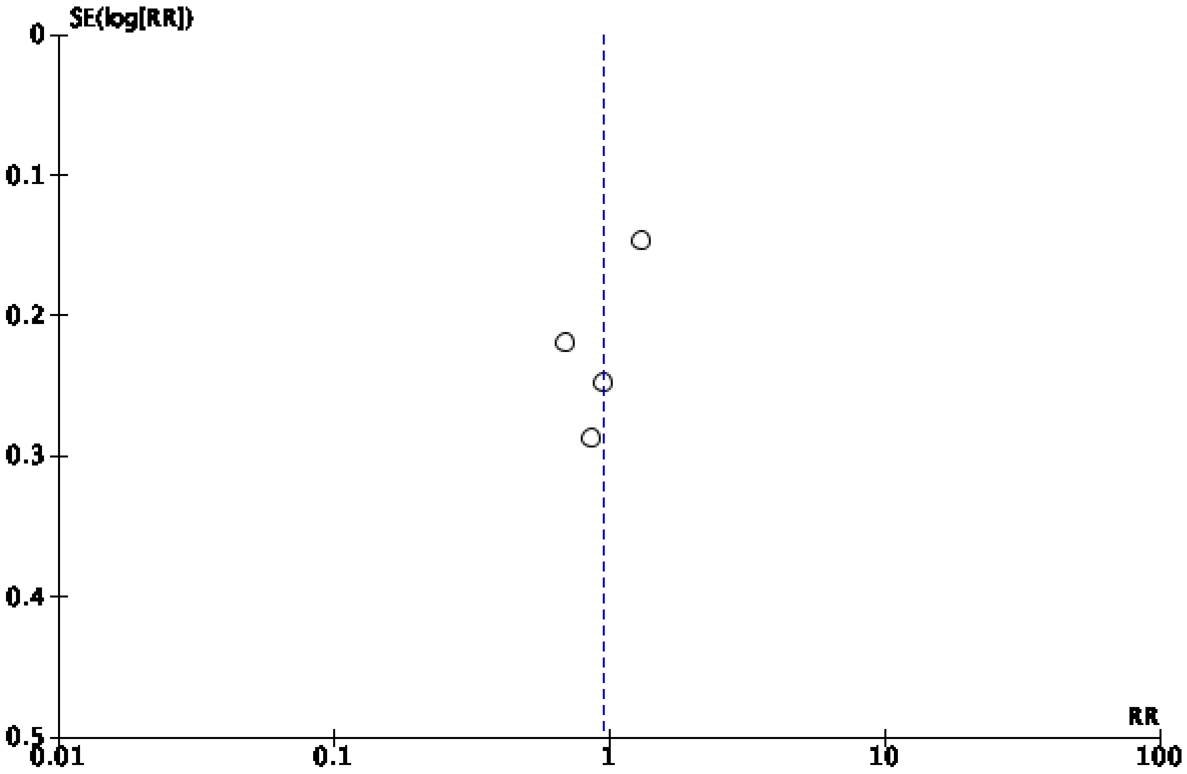

Figure S72. Funnel plot of comparison: Infections.

Protein: Mortality

Study or Subgroup	Higher Protein Events	Higher Protein Total	Lower protein Events	Lower protein Total	Weight	Risk Ratio M-H, Random, 95% CI	Year
Beale 2008	5	27	3	28	2.6%	1.73 [0.46, 6.54]	2008
Doig 2015	42	236	47	235	33.0%	0.89 [0.61, 1.29]	2015
Ferrie 2016	12	59	9	60	7.5%	1.36 [0.62, 2.98]	2016
Wischmeyer 2017	8	52	17	73	8.0%	0.66 [0.31, 1.41]	2017
Fetterplace 2018	4	30	5	30	3.1%	0.80 [0.24, 2.69]	2018
Ridley 2018	19	51	13	48	13.5%	1.38 [0.77, 2.47]	2018
van Zanten 2018	3	22	3	22	2.1%	1.00 [0.23, 4.42]	2018
Zhu 2018	25	176	40	189	22.3%	0.67 [0.43, 1.06]	2018
Nakamura 2019	4	26	5	24	3.3%	0.74 [0.22, 2.43]	2019
Berger 2019	0	11	1	12	0.5%	0.36 [0.02, 8.04]	2019
Nakamura 2021	6	60	6	57	4.0%	0.95 [0.33, 2.78]	2021
Total (95% CI)		750		778	100.0%	0.90 [0.73, 1.12]	
Total events	128		149				

Heterogeneity: Tau² = 0.00; Chi² = 6.73, df = 10 (P = 0.75); I² = 0%
Test for overall effect: Z = 0.95 (P = 0.34)

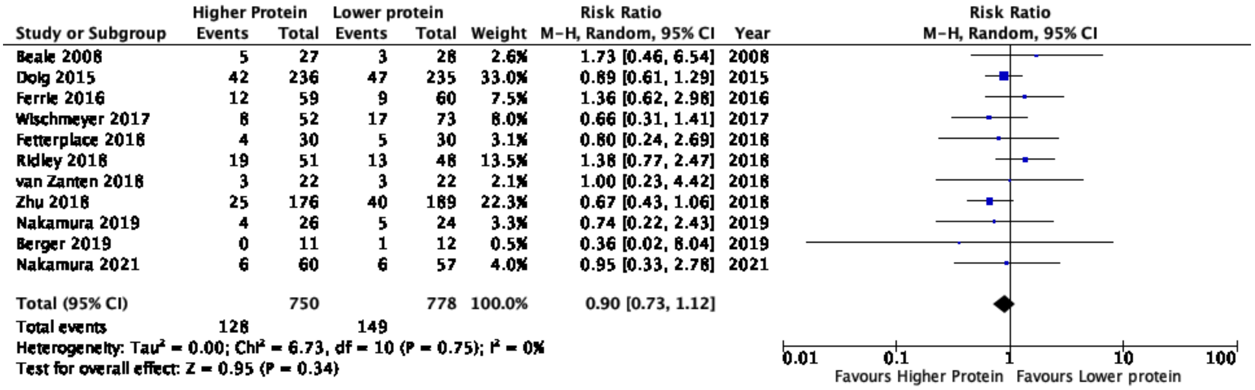

Figure S73. Forest plot of comparison: Mortality.

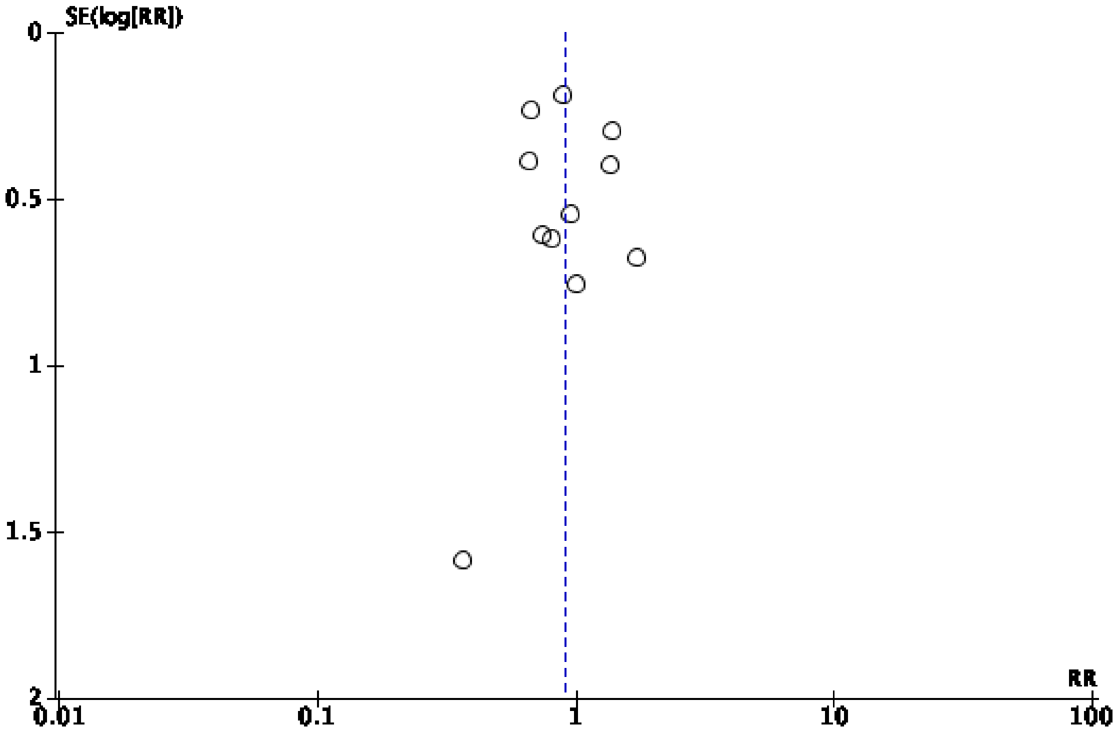

Figure S74. Funnel plot of comparison: Mortality.

Protein: Length of hospital stay

Study or Subgroup	Higher Protein Mean	Higher Protein SD	Higher Protein Total	Lower protein Mean	Lower protein SD	Lower protein Total	Weight	Mean Difference IV, Random, 95% CI	Year
Reilly 1990	44	25	10	47.2	19	10	0.5%	−3.20 [−22.66, 16.26]	1990
Beale 2008	43.8	36.6	27	31.3	27.2	28	0.6%	12.50 [−4.59, 29.59]	2008
Doig 2015	26	2.8	239	24.8	2.7	235	33.1%	1.20 [0.70, 1.70]	2015
Ferrie 2016	25	18.1	59	27.5	27.4	60	2.4%	−2.50 [−10.83, 5.83]	2016
Jakob 2017	31	5.9	46	36	9	44	11.9%	−5.00 [−8.16, −1.84]	2017
Wischmeyer 2017	23.5	12.7	52	24	16.5	73	5.7%	−0.50 [−5.62, 4.62]	2017
Fetterplace 2018	27.4	19	30	18.8	10.9	30	2.7%	8.60 [0.76, 16.44]	2018
Ridley 2018	22	21	51	23	17	48	2.9%	−1.00 [−8.51, 6.51]	2018
van Zanten 2018	28.5	13.3	22	28.2	13.2	22	2.7%	0.30 [−7.53, 8.13]	2018
Zhu 2018	25.3	3.1	179	23.7	2.9	189	32.3%	1.60 [0.99, 2.21]	2018
Nakamura 2019	20.4	10.7	26	19.8	12.5	24	3.8%	0.60 [−5.87, 7.07]	2019
Berger 2019	44	20	11	48	25	12	0.5%	−4.00 [−22.43, 14.43]	2019
Nakamura 2021	26.5	29.6	60	45.5	50	57	0.8%	−19.00 [−33.99, −4.01]	2021
Total (95% CI)			812			832	100.0%	0.36 [−0.98, 1.70]	

Heterogeneity: $Tau^2 = 1.35$; $Chi^2 = 30.56$, df = 12 ($P = 0.002$); $I^2 = 61\%$
Test for overall effect: Z = 0.52 (P = 0.60)

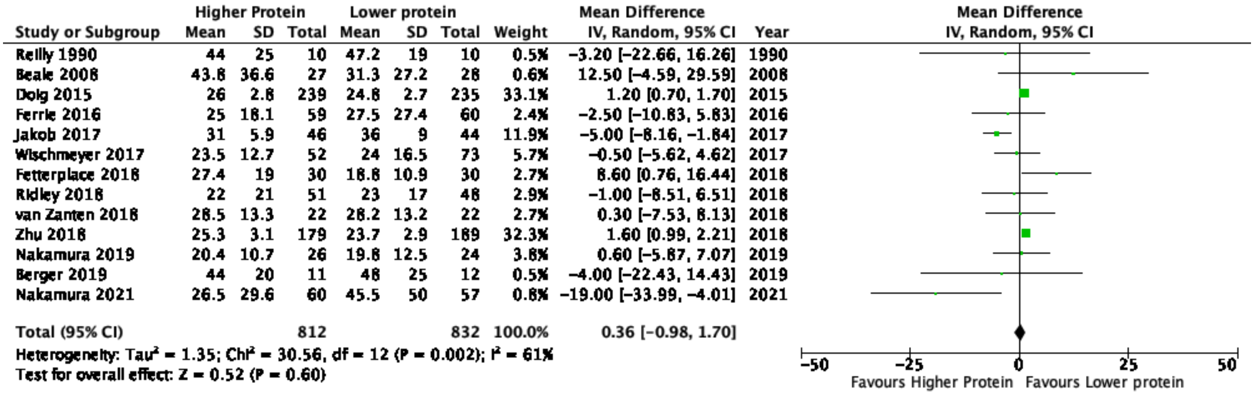

Figure S75. Forest plot of comparison: Length of hospital stay.

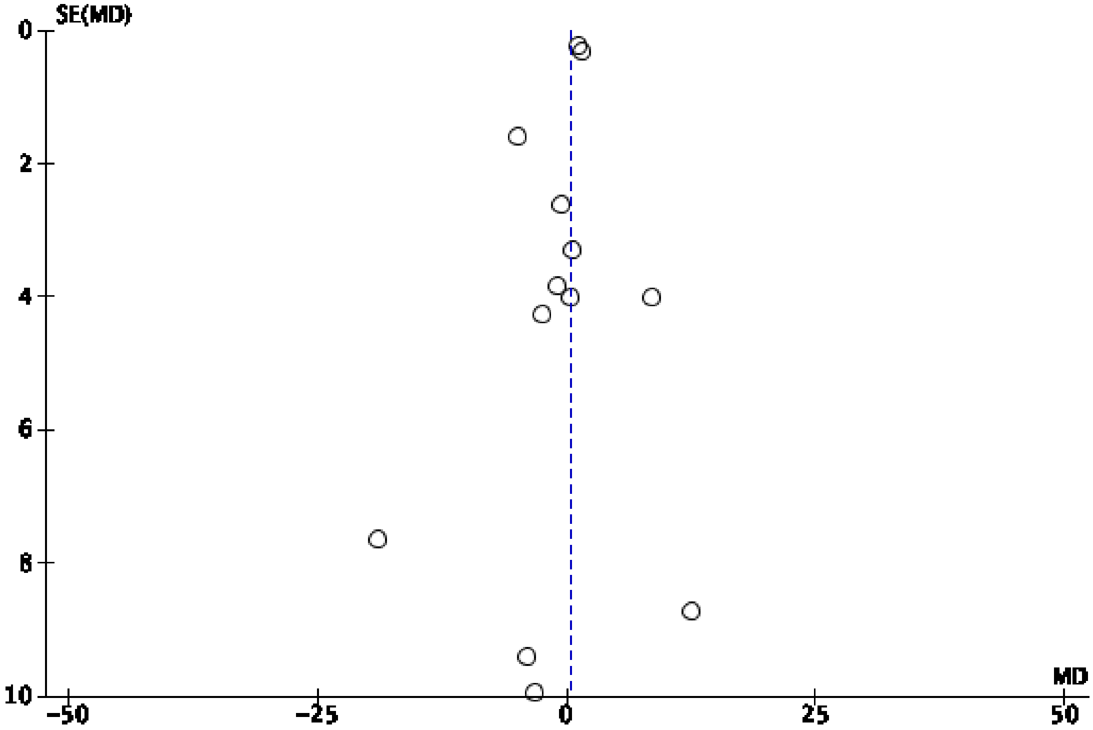

Figure S76. Funnel plot of comparison: Length of hospital stay.

Certainty assessment							№ of patients		Effect		Certainty	Importance
№ of studies	Study design	Risk of bias	Inconsistency	Indirectness	Imprecision	Other considerations	[Intervention]	[Control]	Relative (95% CI)	Absolute (95% CI)		
**ADL score**												
0	randomised trials								-	**0** (0 to 0)	-	CRITICAL
**Hand grip strength（kg)**												
2	randomised trials	serious[a]	not serious	not serious	very serious[b]	none	93	99	-	**SMD 0.07 higher** (0.22 lower to 0.35 higher)	⨁◯◯◯ Very low	CRITICAL
**Arm muscle area, %**												
1	randomised trials	serious[a]	not serious	not serious	very serious[c]	none	74	75	-	MD **0.4 lower** (3.72 lower to 2.92 higher)	⨁◯◯◯ Very low	CRITICAL
**QOL: ED-5D-3L**												
2	randomised trials	very serious[d]	not serious	not serious	very serious[b]	none	263	288	-	MD **0.01 higher** (0.03 lower to 0.05 higher)	⨁◯◯◯ Very low	CRITICAL
**Diarrhea**												
3	randomised trials	not serious	serious[e]	not serious	very serious[f]	none	121/566 (21.4%)	107/554 (19.3%)	**RR 0.85** (0.30 to 2.47)	**33 more per 1,000** (from 141 fewer to 292 more)	⨁◯◯◯ Very low	CRITICAL
**Mortality**												
8	randomised trials	not serious	not serious	not serious	serious[g]	none	341/1369 (24.9%)	341/1385 (24.6%)	**RR 1.00** (0.88 to 1.14)	**0 fewer per 1,000** (from 30 fewer to 34 more)	⨁⨁⨁◯ Moderate	IMPORTANT
**Hospital day**												
7	randomised trials	not serious	serious[h]	not serious	very serious[f]	none	401	391	-	MD **1.08 lower** (4.86 lower to 2.7 higher)	⨁◯◯◯ Very low	IMPORTANT

CI: confidence interval; **MD:** mean difference; **RR:** risk ratio; **SMD:** standardised mean difference

Explanations

a. Downgraded one level due to high risk of bias (difficulty in blinding patients and providers, incomplete outcomes)

b. Many have small sample sizes where the 95% CI includes no effect and does not meet OIS

c. One RCT, and many with small sample sizes where the 95% CI includes no effect and does not meet OIS

d. Downgraded 2 levels due to subjective outcome and very high risk of bias (difficulty in blinding patients and providers, unknown blinding of assessors, incomplete outcome)

e. I2=55%, one step downgrade

f. 95% CI not including no effect, less than OIS

g. Meets OIS but 95% CI includes no effect

h. I2=45%, one step downgrade

Figure S77. Evidence profile about optimal energy delivery.

Certainty assessment							№ of patients		Effect		Certainty	Importance
**№ of studies**	**Study design**	**Risk of bias**	**Inconsistency**	**Indirectness**	**Imprecision**	**Other considerations**	**Intervention**	**Control**	**Relative (95% CI)**	**Absolute (95% CI)**		
**Barthel index**												
3	randomised trials	not serious	serious[a]	not serious	serious[b]	none	114	122	-	MD **21.55 higher** (1.3 lower to 44.4 higher)	⊕⊕◯◯ *Low*	CRITICAL
**hand grip strength**												
2	randomised trials	serious[c]	not serious	not serious	serious[d]	none	25	40	-	MD **1 lower** (5.79 lower to 3.79 higher)	⊕⊕◯◯ *Low*	CRITICAL
**muscle volume change**												
3	randomised trials	not serious	not serious	not serious	serious[e]	none	145	141	-	SMD **0.47 higher** (0.24 higher to 0.71 higher)	⊕⊕⊕◯ *Moderate*	CRITICAL
**QOL score (physical)**												
3	randomised trials	serious[f]	not serious	not serious	serious[g]	none	361	352	-	SMD **0.13 lower** (0.31 lower to 0.06 higher)	⊕⊕◯◯ *Low*	CRITICAL
**Diarrhea**												
7	randomised trials	not serious	serious[h]	not serious	serious[i]	none	93/234 (39.7%)	104/231 (45.0%)	**RR 0.90 (0.61 to 1.31)**	**45 fewer per 1,000** (from 176 fewer to 140 more)	⊕⊕◯◯ *Low*	CRITICAL
**Mortality**												
11	randomised trials	not serious	not serious	not serious	serious[j]	none	128/750 (17.1%)	149/778 (19.2%)	**RR 0.90 (0.73 to 1.12)**	**19 fewer per 1,000** (from 52 fewer to 23 more)	⊕⊕⊕◯ *Moderate*	IMPORTANT
**hospital day**												
13	randomised trials	not serious	serious[k]	not serious	not serious	none	812	832	-	MD **0.36 higher** (0.98 lower to 1.7 higher)	⊕⊕⊕◯ *Moderate*	IMPORTANT

CI: confidence interval; **MD:** mean difference; **RR:** risk ratio; **SMD:** standardised mean difference

Explanations

a. I2 is high at 76%. However, the forest plot was the same direction. Therefore, downgrage was limitted to serious.

b. Downgraded due to total sample size of 236<800

c. Included two studies have high risk of bias about blinding of participants and personael and incomplete outcome data.

d. Downgraded due to total sample size of 65<800

e. Downgraded due to total sample size of 286<800

f. No study was judged to be low risk of bias about blinding of participants and personael and blinding of outcome assessment.

g. Downgraded due to total sample size of 712<800

h. I2 is high at 64%. Forest plot direction a little bit different among included studies.

i. Downgraded due to total events size of 197<300

j. Downgraded due to total events size of 277<300

k. I2 is high at 61%.

Figure 78. Evidence profile about optimal protein delivery.
